# Signal peptide cleavage and ectodomain regions of GP2 are required for PRRSV infection

**DOI:** 10.1099/jgv.0.002287

**Published:** 2026-06-18

**Authors:** Derek Pinto, Raymond R. R. Rowland, Alberto Brandariz-Nuñez

**Affiliations:** 1 Department of Pathobiology, College of Veterinary Medicine, University of Illinois at Urbana-Champaign, Champaign, IL, USA

**Keywords:** CD163, GP2, PRRSV, signal peptide, viral glycoprotein

## Abstract

Porcine reproductive and respiratory syndrome virus (PRRSV) is the most economically important pathogen of swine, yet the molecular mechanisms governing its entry into host cells remain incompletely understood. The minor envelope glycoprotein GP2, together with GP3 and GP4, forms an essential complex that engages the entry receptor CD163; however, the specific GP2 regions required for receptor association have not been fully defined experimentally. Here, we investigated GP2 processing, intracellular trafficking and interactions with viral glycoproteins and CD163. We demonstrate that the GP2 signal peptide (SP) is cleaved in both transfected and infected cells and is necessary and sufficient for ER localization. Removal of the SP disrupted GP2 maturation, impaired interactions with GP3, GP4 and GP5, and significantly reduced viral infectivity in infectious clone assays. Deletion of the SP also abolished GP2–CD163 association, indicating that proper SP-dependent processing is required for receptor engagement. Using co-immunoprecipitation and colocalization analyses, we identified two highly conserved regions within the GP2 ectodomain that associate with CD163. Deletion of either region completely eliminated PRRSV infection. Together, these findings define the structural determinants within GP2 required for association with CD163 and advance our understanding of the early steps of PRRSV entry.

## Data availability

No new nucleotide or amino acid sequence data were generated in this study. All sequences used for alignment analyses were obtained from publicly available databases (GenBank), and the corresponding accession numbers are provided in the manuscript.

## Introduction

Porcine reproductive and respiratory syndrome virus (PRRSV) is endemic in most swine-producing countries and remains the most economically devastating pathogen affecting global pork production [1]. PRRSV is an enveloped, positive-sense single-stranded RNA virus belonging to the genus *Betaarterivirus* within the family *Arteriviridae* [2,4]. Two viral species are recognized: Betaarterivirus suid 1 (PRRSV-1) and Betaarterivirus suid 2 (PRRSV-2) [5], which share only ~70% nucleotide identity [6], yet both circulate in U.S. herds and cause substantial economic losses [1,7]. PRRSV exhibits a strong tropism for CD163-expressing macrophages, particularly porcine alveolar macrophages (PAMs), which serve as the principal target cells *in vivo* [8]. CD163, a scavenger receptor expressed exclusively on cells of the monocyte – macrophage lineage [9,10], is the essential entry receptor for PRRSV [11,12]. The role of CD163 as the essential viral receptor for PRRSV was supported by studies in genetically modified pigs showing that CD163 is both necessary and sufficient for PRRSV infection [12,15]. Although the exact molecular functions of CD163 remain incompletely understood, it has been proposed that CD163 cooperates with CD169 to facilitate viral internalization [12,14]. CD163 has also been implicated in mediating viral membrane fusion and uncoating within target cells [12,14,16].

**Fig. 1.**
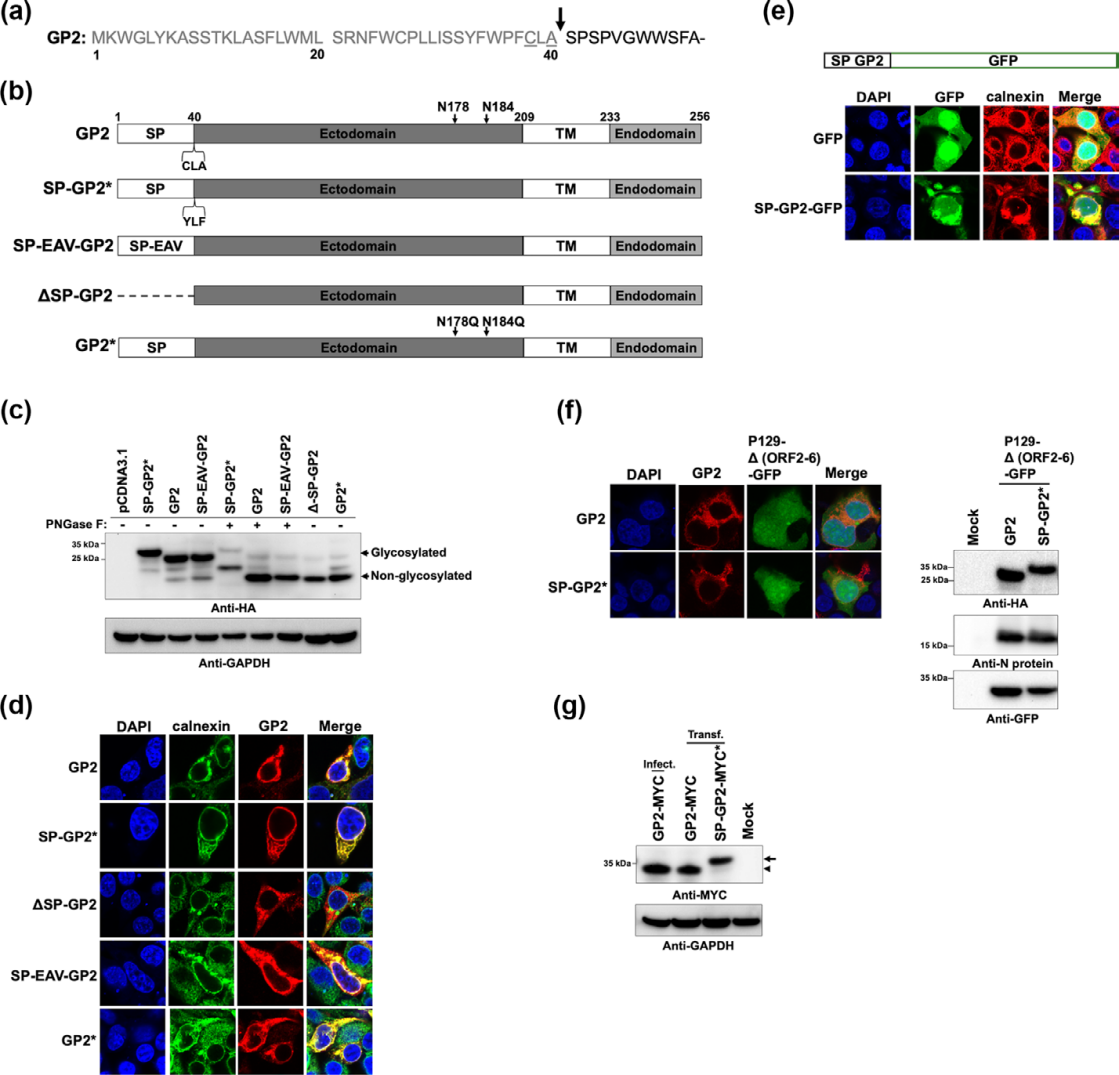
Characterization of the GP2 SP and its role in protein processing and subcellular localization. (a) Prediction of SP within the GP2 amino acid sequence. The first 51 amino acids of GP2 from the PRRSV-2 isolate P129 are shown. The SP cleavage site with the highest probability, as predicted by SignalP, is indicated by an arrow. The N-terminal SP region is shaded in grey. The −1 and −3 residues recognized by signal peptidase are underlined. The SignalP-predicted d-score for cleavage at the indicated site is 0.925 (cutoff=0.45). A similar SP prediction was obtained using Phobius and PolyPhobius [44,45]. (**b**) Schematic representation of GP2 mutants. The predicted SP, ectodomain, transmembrane region (TM) and endodomain of GP2 are shown as rectangles, with their respective lengths indicated above. TM domain was predicted using Phobius and PolyPhobius [44,45]. The *N*-glycosylation sites are also shown. Dotted lines indicate deleted amino acid regions. In the SP-GP2* mutant, the small residues C38 and A40 were substituted with Y and F, respectively. For the *N*-glycosylation-deficient mutant (GP2*), the *N*-glycosylation sites were replaced with glutamine (**q**) residues. (**c**) Deletion of the GP2 SP generates a variant that is not *N*-glycosylated, whereas the SP of GP2 is efficiently cleaved in transfected cells. Western blot (WB) analysis of GP2 variant expression levels in transfected cells. HEK293T cells were transfected with plasmids expressing the indicated GP2 mutants containing a C-terminal hemagglutinin (HA) tag. Cell lysates were either left untreated or digested with PNGase F and then immunoblotted with an anti-HA antibody. GAPDH was used as a loading control. (**d**) Colocalization of GP2 variants with the ER. HEK293T cells were transfected with plasmids expressing the indicated GP2 mutants containing a C-terminal HA tag. After 24 h of incubation, cells were fixed and stained with an anti-HA antibody (red). The ER was visualized using an anti-calnexin antibody (green). Nuclei were counterstained with DAPI (blue). Representative merged images showing colocalization (yellow) are shown. (**e**) Fusion of the GP2 SP to GFP induces localization of GFP to the ER. HEK293T cells were transfected with plasmids expressing either GFP alone or a fusion protein containing the GP2 SP fused to GFP. After 24 h of incubation, cells were fixed and the ER was visualized using an anti-calnexin antibody (red). Nuclei were counterstained with DAPI (blue). GFP fluorescence was visualized directly without antibody staining. Representative merged images showing colocalization (yellow) are shown. (**f**) The GP2 SP is efficiently cleaved when GP2 is co-expressed with a noninfectious PRRSV clone lacking ORFs 2–6 and expressing GFP (P129-Δ(ORF2–6)-GFP). Left panels: HEK293T cells were co-transfected with plasmids expressing the indicated GP2 variants containing a C-terminal HA tag together with P129-Δ(ORF2-6)-GFP. At 24 h post-transfection, cells were fixed and GP2 variants were immunostained as indicated above. Nuclei were counterstained with DAPI (blue). GFP fluorescence was visualized directly without antibody staining. Right panels: HEK293T cells were co-transfected with the same plasmids, and cell lysates were analysed by WB using anti-HA, anti-GFP and anti-N monoclonal antibodies. (**g**) The GP2 SP is efficiently cleaved in infected cells expressing GP2-MYC. MARC-145 cells were transfected with plasmids expressing GP2-MYC or SP-GP2-MYC* and analysed by WB using an anti-MYC antibody (Transf.). GP2-MYC from cells infected with an infectious clone expressing GP2-MYC was also detected by WB with the anti-MYC antibody (Infect.). GP2-MYC* harbouring mutations that prevent SP cleavage is indicated with a long arrow, whereas GP2-MYC from either transfected or infected cells is indicated with a short arrow on the right side of the WB panel.

The ~15 kb PRRSV genome contains at least ten open reading frames encoding the structural proteins and nonstructural proteins nsp1–12 [3,17]. Structural proteins include four glycosylated envelope glycoproteins – GP2, GP3, GP4 and GP5 – and three non-glycosylated envelope proteins – E, ORF5a and M – as well as the nucleocapsid protein N [18,19]. GP5 and M are the major envelope components and form a disulfide-linked GP5–M heterodimer on the virion surface [20,21]. In contrast, GP2, GP3 and GP4 form a non-covalently associated heterotrimer [20,22], which plays an essential role in viral entry and infectivity [16,23]. GP2 (also referred to as GP2a) is a type I integral membrane protein [20,24] that contains an N-terminal signal peptide (SP), an ectodomain, a membrane-spanning region, a C-terminal intracellular tail and two predicted *N*-glycosylation sites. A schematic representation of PRRSV-2 GP2 is shown in Fig. 1b.

The GP2–GP3–GP4 complex contains multiple conserved *N*-glycosylation sites that are essential for infectious virus production and viral infectivity [23,25]. The loss of infectivity observed after removal of specific *N*-glycan residues is likely due to the requirement of these glycans for proper protein folding and stability, which are necessary for efficient virion assembly [26]. Importantly, removal of *N*-glycosylation from these glycoproteins does not impair their ability to interact with CD163, indicating that the essential contribution of these glycans lies primarily in maintaining structural integrity rather than mediating receptor engagement [26]. However, the specific role of *N*-glycosylation in promoting or stabilizing the GP2–GP3–GP4 heterotrimer remains unclear.

The GP2–GP3–GP4 complex, together with GP5/M, can associate with CD163, facilitating viral attachment and internalization [16,27,29]. However, the precise regions within the viral glycoproteins that mediate CD163 binding remain poorly defined. The tropism of PRRSV is influenced by the minor structural proteins E, GP2, GP3 and GP4 [30,31], and substitutions in GP2 residues 91, 97 and 98 critically affect PRRSV-2 replication and Marc-145 cell adaptation [32,33]. Additional studies suggest that the GP2 160^th^ amino acid also contributes to PRRSV-2 cell tropism [34], and host factors, such as DDX27 and NLRP12, restrict PRRSV by promoting GP2 degradation through autophagy- or ubiquitin-mediated pathways [35,36]. Furthermore, GP2–GP4 influence the spread pattern and viral yield of PRRSV-2 in Marc-145 cells [37]. Collectively, these findings underscore GP2 as a key determinant of PRRSV tropism, replication efficiency and susceptibility to host antiviral responses.

This study aims to define the specific regions of GP2 that are required for association with CD163. We first examined GP2 processing and demonstrated that its SP is cleaved in both transfected and infected cells and is necessary and sufficient for ER localization. We further show that the SP is required for efficient GP2 interaction with GP3, GP4 and GP5, consistent with early co-assembly of these glycoproteins in the ER and that SP mutations impair viral infection. We also found that removal of *N*-glycosylation does not affect the association of GP2 with any of the other viral glycoproteins. Deletion of the SP disrupts GP2–CD163 association, likely due to defective folding and maturation. Finally, co-immunoprecipitation (co-IP) and colocalization analyses allowed us to map two highly conserved regions within the GP2 ectodomain that associate with CD163, and we show that deletion of either region abolishes PRRSV infection.

## Methods

### Plasmids

The plasmid pCDNA3.1-CD163-FLAG, which expresses pig CD163 (GenBank accession number: NM_213976.1) fused to a FLAG epitope, was purchased from GenScript (Piscataway, NJ, USA). The construction of the recombinant plasmid, pCMV-HA-C-GP2, which expresses the viral GP2 from P129 strain [38] fused to a HA epitope, has been described previously [28]. Plasmids expressing GFP fused to the SP of PRRSV GP2, as well as GP2 containing a replacement of its native SP with that of EAV GP2 (GenBank accession number: AYF58785.1), were generated by synthesizing the corresponding DNA sequences flanked by EcoRI and XhoI restriction sites (Integrated DNA Technologies (IDT), Coralville, IA, USA). The synthesized fragments were cloned into the EcoRI and XhoI sites of the pCMV-HA-C vector. The plasmid pCMV-HA-C-GP2, the Q5 site-directed mutagenesis kit (New England Biolabs, Ipswich, MA, USA) and specific primers (Table S1, available in the online Supplementary Material) were used according to the manufacturer’s instructions to generate recombinant plasmids expressing HA-tagged GP2 deletion and point mutants. Constructs not listed in Table S1 were synthesized by Twist Bioscience (South San Francisco, CA, USA). Recombinant plasmids expressing GP2 fused to a MYC epitope were generated using pCMV-HA-C-GP2 as a template, the Q5 site-directed mutagenesis kit (New England Biolabs, Ipswich, MA, USA) and specific primers (Table S1). The plasmid expressing SP-GP2-MYC* was generated using the GP2-MYC plasmid as a template, following the same procedure. Recombinant plasmids expressing the viral GP4 and GP5 proteins from the P129 strain [38], fused to V5 and FLAG epitopes, respectively, were generated using pCMV-HA-C-GP4 and pCMV-HA-C-GP5 as templates [28], the Q5 site-directed mutagenesis kit (New England Biolabs, Ipswich, MA, USA) and specific primers (Table S1). To express GP2 fused to the N terminus of GFP, the recombinant plasmid pCMV-HA-C-GP2 was used as a template for PCR amplification with specific primers (forward: 5′-TAAGGCCTCTCTCGAGGCCACCATGAAATGGGGTCCATGCAAAGCC-3′; reverse: 5′-GTCGACTGCAGAATTCTCTGTGAGTTCGAAAGAAAAATTGC-3′). The PCR product was digested and cloned into the XhoI and EcoRI sites of the pEGFP-N1 vector. To express GP3 fused to a C-terminal MYC tag, the recombinant plasmid pCMV-HA-C-GP3 was used as a template for PCR amplification with specific primers (forward: 5′-TAAGGCCTCTGAATTCGCCACCATGGTTAATAGCTGTACATTC-3′; reverse: 5′-CAGAATACGTCTCGAGTCAGAGGTCTTCTTCCGATATCAATTTCTGTTCTCGCCGTACGGCACTGAGGG-3′). The PCR product was digested and cloned into the EcoRI and XhoI sites of the pCMV-HA-C vector. The recombinant plasmid expressing an HA-tagged GP2 mutant in which all *N*-linked glycosylation sites were substituted with glutamine (Q) was previously described [26]. Recombinant plasmids expressing the *N*-glycosylation–deficient mutants GP3-MYC*, GP4-V5* and GP5-FLAG* were generated by substituting the *N*-linked glycosylation sites with glutamine (Q) residues, as previously described [26]. The correctness of all constructs was verified by sequencing and by Western blot (WB) analysis of the expressed proteins.

### Generation of mutant GP2 PRRSV genomes

The full-length infectious cDNA clone of the PRRSV P129 strain expressing GFP [39], derived from the P129 isolate (GenBank accession no. AF494042.1), was used as the backbone for introducing mutations into the viral envelope glycoprotein GP2. Infectious clones expressing a MYC-tagged GP2, as well as clones harbouring specific GP2 mutations, were synthesized by Twist Bioscience. All synthesized clones were sequence-verified to confirm the presence of the intended GP2 mutations in the full-length genomic cDNA.

### Viruses and cells

HEK293T cells (ATCC CRL-3216; American Type Culture Collection, Rockville, MD) and African green monkey kidney cells MARC-145 (RRID:CVCL_4540) were cultured at 37 °C in 5% CO₂ in Dulbecco’s modified Eagle’s medium (DMEM; Life Technologies) supplemented with 10% FBS(Gibco), 2 mM l-glutamine (Life Technologies) and antibiotics (100 U ml^−1^ penicillin and 100 µg ml^−1^ streptomycin; Life Technologies). The PRRSV-2 isolate, a P129 strain expressing GFP [39], was propagated and titrated on MARC-145 cells as previously described [29]. P129 is a virulent PRRSV strain isolated in 1995 from an outbreak of highly pathogenic PRRSV in southern Indiana, USA [38].

### Antibodies and reagents

The monoclonal anti-FLAG M2 antibody was purchased from Sigma. Rabbit monoclonal antibodies against FLAG tag (D6W5B) and HA tag (C29F4) and a mouse monoclonal antibody against HA tag (6E2) were obtained from Cell Signalling Technology. The PRRSV nucleocapsid antibody used for WB was obtained from Novus Biologicals. Alexa Fluor 647–conjugated anti-rabbit IgG, Alexa Fluor 568–conjugated anti-mouse IgG and Alexa Fluor 594– and 488–conjugated secondary antibodies against mouse and rabbit IgG, respectively, were from Thermo Fisher Scientific. The anti-GFP antibody was purchased from R and D Systems. Anti-FLAG agarose beads and 3×FLAG peptide were from Sigma, while anti-HA agarose beads and HA peptide were from Thermo Fisher Scientific. The following antibodies were also obtained from Cell Signalling Technology: anti-GAPDH (D4C6R) monoclonal antibody, calnexin (C5C9) rabbit monoclonal antibody, anti-MYC mouse monoclonal antibody (9B11), anti-MYC rabbit monoclonal antibody (71D10) and anti-V5 rabbit monoclonal antibody (D3H8Q).

### Treatment with *N*-glycosylation glycosidases

Prior to enzymatic treatment, cell lysates were mixed with Glycoprotein Denaturing Buffer (New England Biolabs) and incubated at 100 °C for 10 min. To remove high-mannose oligosaccharides, denatured lysates were treated with 2,500 units of endoglycosidase H (Endo H; New England Biolabs) for 1 h at 37 °C. Peptide-N-glycosidase F (PNGase F; New England Biolabs) was used to remove all N-linked oligosaccharides under the same conditions (1 h at 37 °C). Deglycosylated proteins were analysed by WB using an anti-HA monoclonal antibody (Cell Signalling Technology).

### Transfection and immunofluorescence microscopy

Cell monolayers were transfected using TransIT-LT1 transfection reagent (Mirus) according to the manufacturer’s instructions. Transfected cells were incubated at 37 °C for 24 h. For indirect immunofluorescence microscopy, cell monolayers grown on coverslips were transfected as indicated in the figure legends. At 24 h post-transfection, cells were fixed with 4% paraformaldehyde, permeabilized with 0.5% Triton X-100 in PBS and blocked with PBS containing 2% bovine serum albumin (Sigma). Cells were then incubated with primary antibodies overnight at 4 °C, followed by incubation with fluorophore-conjugated secondary antibodies and DAPI for 1 h at room temperature. Images were acquired using a Nikon A1R laser scanning confocal microscope and processed with NIS-Elements software (Nikon) [40].

### Virus preparation from full-length cDNA clones

HEK293T cells were transfected with the full-length PRRSV cDNA plasmid using TransIT-LT1 transfection reagent (Mirus) according to the manufacturer’s instructions. Transfected cells were maintained in DMEM supplemented with 10% FBS for 48 h. The supernatant from transfected cells was then used to infect MARC-145 cells, which were incubated for 3 days. The resulting virus-containing supernatant was designated as ‘passage 1’. Passage 1 virus was used to inoculate fresh MARC-145 cells, and the 3 day harvest was designated as ‘passage 2’. Passage 3 virus was prepared in the same manner. Each virus passage was aliquoted and stored at −80 °C until use.

### Western blotting

Cellular proteins were extracted using whole-cell extract (WCE) buffer (50 mM Tris-HCl, pH 7.5; 150 mM NaCl; 0.5% Triton X-100; 10% glycerol; 1 mM EDTA) supplemented with a protease inhibitor cocktail (Sigma), as previously described [40]. Cell lysates were resolved on 4–12% Bis-Tris NuPAGE gels (Invitrogen) and transferred to nitrocellulose membranes using a Trans-Blot Turbo transfer system (Bio-Rad). Protein detection was performed using specific primary antibodies, followed by HRP-conjugated secondary antibodies against rabbit or mouse (Cell Signalling Technology). Immunoreactive bands were visualized using SuperSignal West Femto Maximum Sensitivity Substrate (Thermo Fisher) and imaged with a FluorChem R system (ProteinSimple).

### Immunoprecipitation assay

Immunoprecipitation (IP) assays were performed as previously described [28,29, 40]. HEK293T cells were co-transfected with plasmids encoding FLAG-tagged CD163 and HA-tagged GP2 variants. At 24 h post-transfection, cells were lysed in WCE buffer supplemented with protease inhibitor cocktail (Sigma). Lysates were cleared by centrifugation (14,000 r.p.m., 4 °C), and a small aliquot of each supernatant was retained as input. Cleared lysates were pre-cleared with protein A-agarose (Sigma) for 1 h at 4 °C with gentle rotation, then incubated overnight at 4 °C with anti-FLAG agarose (Sigma) to capture FLAG-tagged proteins. Beads were washed three times with WCE buffer, and immune complexes were eluted in WCE buffer (without Triton X-100) using 200 µg ml^−1^ 3×FLAG peptide. Eluates and inputs were separated by SDS–PAGE and analysed by WB with anti-HA and anti-FLAG antibodies. In reciprocal experiments, HEK293T cells expressing FLAG-CD163 or GP2-HA variants were lysed separately (1 ml WCE buffer per dish), cleared, pre-cleared with protein A-agarose and post-spin lysates were then mixed 1 : 1 prior to incubation with anti-FLAG agarose overnight at 4 °C. Beads were washed four times with WCE, and complexes were eluted with 200 µg ml^−1^ 3×FLAG peptide in WCE buffer lacking Triton X-100. Eluates were analysed by SDS–PAGE and WB as described above. For IPs assessing GP2-HA variant associations with MYC-tagged GP3, V5-tagged GP4 and FLAG-tagged GP5, anti-HA agarose (Thermo Fisher) was used to capture HA-tagged proteins. Beads were washed four times with WCE buffer, and immune complexes were eluted with 1 mg ml^-1^ HA peptide in WCE buffer without Triton X-100. Eluates were analysed by SDS–PAGE and WB with anti-HA, anti-FLAG, anti-MYC and anti-V5 antibodies.

### Quantitation of co-immunoprecipitation efficiency

Input and immunoprecipitated proteins were detected by WB using specific antibodies and visualized as described above. For densitometric analysis, the relative intensities of protein bands were quantified using ImageJ software (version 2.1.0; National Institutes of Health, USA). The co-IP index was defined as the intensity of the HA-tagged protein in the immunoprecipitated fraction divided by the sum of the intensities of the HA-tagged protein in the input and the FLAG-tagged protein in the immunoprecipitated [41]. Co-IP indices of GP2 variants are expressed relative to those of wild-type (WT) GP2.

### Quantification and statistical analysis

Densitometric values were obtained using ImageJ software (version 2.1.0; National Institutes of Health, USA). Data are presented as mean±sd. Statistical analyses were performed using GraphPad Prism 7.0 c. Two-tailed unpaired Student’s t-tests were used, and a *P-*value <0.05 was considered statistically significant.

## Results

### Contribution of the SP of GP2 to protein processing and intracellular trafficking

A recent report characterizing the evolution of Type 2 PRRSV through whole-genome analysis predicted that PRRSV GP2 contains an N-terminal SP spanning amino acids 1–38 [42]. However, there is no experimental evidence confirming whether this SP is cleaved or identifying its exact cleavage position. To determine whether the GP2 SP is cleaved and where the cleavage occurs, we first performed a bioinformatic prediction using SignalP 3.0 [43]. This tool was applied to analyse the GP2 sequence from PRRSV-2 (P129 isolate). The results showed that GP2 contains a 40–amino acid SP, with SignalP predicting a cleavage site between positions 40 and 41 with high confidence (d-score: 0.925) (Fig. 1a). A similar SP prediction was obtained using Phobius and PolyPhobius [44,45].

To analyse GP2 SP cleavage, we generated a series of C-terminal HA-tagged constructs designed to identify the exact cleavage site (Fig. 1b). We compared the SDS–PAGE mobility of WT GP2 from the PRRSV-2 prototype strain P129 [39] expressed in cells with that of mutants for which the presence or absence of the SP was defined. In one mutant (ΔSP-GP2), the entire SP was deleted (Fig. 1b). In another mutant (SP-GP2*), the small residues alanine (−1) and cysteine (−3) at the predicted cleavage site were substituted with the bulkier residues phenylalanine and tyrosine, respectively (Fig. 1b). Small amino acids at these positions are required for efficient recognition and cleavage by signal peptidase [46,48], and accordingly, SignalP predicts that the SP in SP-GP2* is not cleaved. As additional controls, we generated a construct in which the GP2 SP was replaced with the 29–amino acid SP from EAV GP2 (SP-EAV-GP2), as well as an *N*-glycosylation–deficient mutant (GP2*) in which all *N*-linked glycosylation sites were substituted with glutamine residues to confirm the size of the unglycosylated GP2 variant (Fig. 1b). Expression of these constructs followed by WB analysis showed that GP2 and SP-GP2* differ in molecular weight, with SP-GP2 migrating slightly slower than GP2, indicating that the introduced −1/−3 mutations indeed inhibited SP cleavage (Fig. 1c). In addition, the molecular weight of GP2 was substantially larger than that of ΔSP-GP2, demonstrating that the N-terminal residues of GP2 function as a SP: in their absence, GP2 is not translocated into the ER lumen and, therefore, cannot acquire its two *N*-linked glycans. Consistently, the size of ΔSP-GP2 closely matched that of the *N*-glycosylation–deficient mutant (GP2*), confirming the lack of *N*-glycosylation (Fig. 1c). Importantly, deglycosylated GP2 (PNGase F–treated) migrated at the same molecular weight as ΔSP-GP2 and GP2*, but remained smaller than both deglycosylated SP-GP2* and untreated GP2. These results indicate that the WT SP is cleaved under normal cellular conditions. Furthermore, the migration of SP-EAV-GP2 was similar to that of GP2, both before and after PNGase F treatment (Fig. 1c), suggesting that the EAV-derived SP is also cleaved, in agreement with the SignalP prediction of a cleavage site between positions 29 and 30 (d-score: 0.879).

Next, to analyse the role of the GP2 SP in intracellular trafficking, we assessed the subcellular localization of the different GP2 variants by immunofluorescence using an anti-HA antibody and the ER marker calnexin. The results showed that WT GP2 colocalized with the ER, suggesting that the main function of the SP is to direct GP2 to the ER (Fig. 1d). SP-GP2*, in which SP cleavage is prevented, also colocalized with the ER, indicating that blocking SP cleavage does not alter ER localization. Replacement of the GP2 SP with the SP from EAV (SP-EAV-GP2) did not affect ER colocalization, suggesting that the SP can be functionally replaced by its homologue from EAV without altering ER targeting (Fig. 1d). As expected, removal of *N*-glycosylation sites from GP2 did not change its ER localization, as the SP remained intact (Fig. 1d). In contrast, the mutant lacking the SP (ΔSP-GP2) did not colocalize with the ER, confirming that the SP is required for ER targeting (Fig. 1d). These results are consistent with our previous findings that ΔSP-GP2 is deglycosylated (Fig. 1c). To further demonstrate that the SP of GP2 directs its localization to the ER, we generated a construct in which the SP of GP2 was fused to the N-terminus of GFP (Fig. 1e). The intracellular distribution of the chimeric protein was then analysed in transfected cells. Fusion of the SP to GFP resulted in strong colocalization with the ER, whereas GFP alone was distributed diffusely throughout the cytoplasm, confirming that the GP2 SP is sufficient to induce ER localization (Fig. 1e).

We then further studied the processing and intracellular trafficking of GP2-HA in transfected cells. Lysates from cells expressing GP2-HA were either left untreated or treated with PNGase F, which removes all N-linked carbohydrates, or with Endo H, which specifically cleaves immature high-mannose N-linked glycans. SDS-PAGE and WB analysis showed that GP2-HA was completely sensitive to Endo H digestion, indicating that it remains retained in the ER, where glycoproteins carry exclusively Endo H–sensitive oligosaccharides (Fig. S1A). These results are consistent with immunofluorescence data showing strong colocalization of GP2-HA with an ER marker (calnexin) but not with a Golgi marker (Golgin-97) (Fig. S1B). We conclude that GP2-HA localizes to and is retained in the ER. The complete Endo H sensitivity and absence of Endo H–resistant forms further confirm that GP2-HA does not reach the Golgi and is not processed into complex-type *N*-glycans. Altogether, these results demonstrate that the GP2 SP is cleaved and is both necessary and sufficient for ER localization, which is critical for proper *N*-glycosylation and protein maturation.

Next, we sought to determine whether the GP2 SP is cleaved in an infection-like context. Because no antibodies are available that specifically recognize the endogenous GP2 protein, we co-expressed GP2 and SP-GP2* together with a non-infectious clone that expresses GFP but does not produce the structural proteins encoded by ORF2–ORF6 (P129-Δ (ORF2-6)-GFP). Fluorescence microscopy confirmed that each GP2 variant was expressed in GFP-positive cells harbouring the non-infectious clone (Fig. 1f). WB analysis showed a clear difference in molecular weight between GP2 and SP-GP2*, with SP-GP2* migrating slightly slower than WT GP2 (Fig. 1f). This mobility shift indicates that the SP is cleaved from WT GP2 in cells expressing the viral nonstructural proteins and N protein, suggesting that GP2 undergoes signal-peptide cleavage under infection-like cellular conditions. To further confirm that the GP2 SP is cleaved during infection, we introduced a C-terminal MYC tag into GP2 within the infectious GFP-expressing clone (P129-GFP) to enable detection by WB. The modified clone was transfected into HEK-293T cells, and 48 h later, the culture supernatant was used to inoculate MARC-145 cells. Fluorescence microscopy demonstrated that the construct produced infectious virus particles, as indicated by GFP expression (Fig. S2A), and WB confirmed *N*-protein expression (Fig. S2B). This P1 virus, derived from the initial transfection, also expressed GP2-MYC, as shown by immunofluorescence (Fig. S2C) and WB analysis using an anti-MYC antibody (Fig. S2B). Next, we attempted to generate a virus carrying the same SP-blocking mutations that prevent signal-peptide cleavage in GP2-MYC. However, these mutations abolished virus production, indicating that preventing SP cleavage is incompatible with viral replication (See next sections). To determine whether the SP is cleaved during infection, we compared the migration of GP2-MYC from infected cells with GP2-MYC expressed in transfected cells, along with the non-cleavable SP-GP2-MYC* mutant. The results showed a clear molecular-weight difference between WT GP2-MYC and SP-GP2-MYC*, with SP-GP2-MYC* migrating slightly more slowly (Fig. 1g). In contrast, GP2-MYC from infected and transfected cells migrated identically (Fig. 1g). Together, these findings suggest that the WT GP2 SP is cleaved during infection.

### Impact of the GP2 SP on GP2–CD163 Interaction

**Fig. 2.**
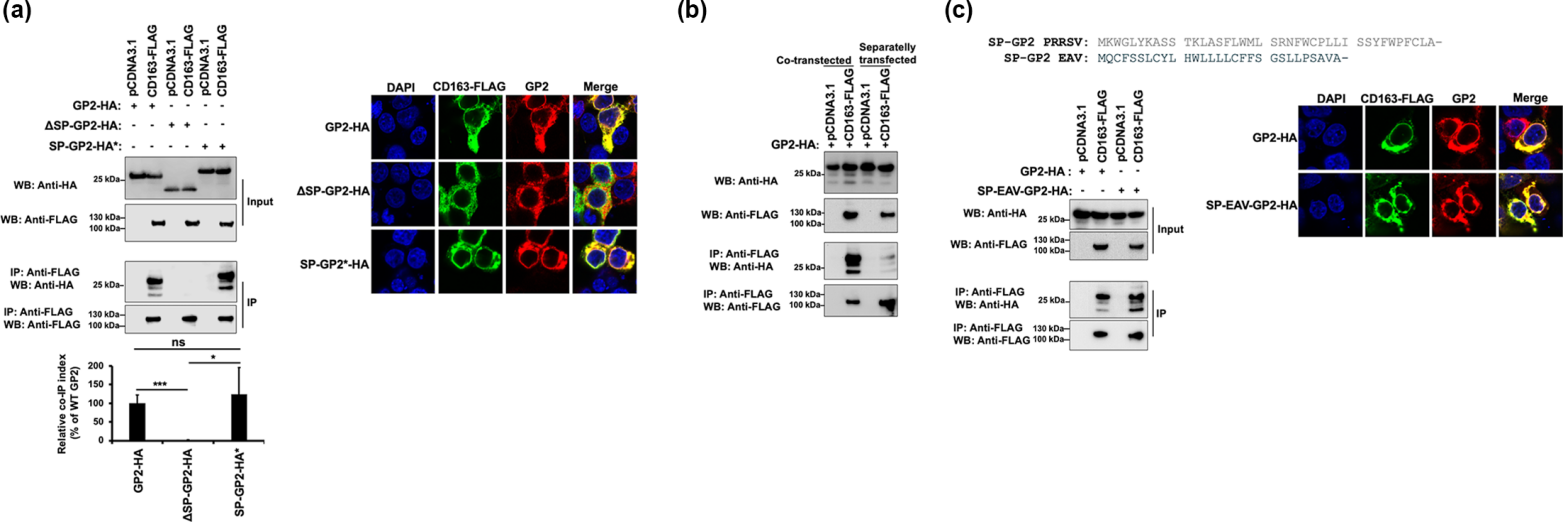
Effect of the GP2 SP on interaction with CD163. (**a**) Co-IP and colocalization analysis of GP2 variants with CD163. HEK293T cells were co-transfected with plasmids expressing WT or mutant GP2-HA and CD163-FLAG. At 24 h post-transfection, cell lysates were analysed by WB with anti-HA and anti-FLAG antibodies (Input) and subjected to IP using anti-FLAG agarose beads. To control for background binding of GP2 variants to anti-FLAG beads, cells were co-transfected with GP2-HA constructs and empty pCDNA3.1. FLAG-peptide eluates were analysed by WB with anti-HA and anti-FLAG antibodies (IP). Band intensities were quantified using ImageJ software, and a co-IP index was calculated for each sample as described in Methods. Quantification represents the mean±sd of three independent experiments. Statistical significance was determined as described in Methods (*P<0.05; ***P<0.001; ns, not significant, *P*≥0.05). Right panels: Colocalization of GP2 variants and CD163. HEK293T cells were transfected with the plasmids expressing the proteins indicated in the figure and, after 24 h, the cells were fixed and immunostained with anti-FLAG (green) and anti-HA (red) antibodies; nuclei were counterstained with DAPI (blue). Representative merged images showing colocalization (yellow) are shown. (**b**) Co-immunoprecipitation of GP2 and CD163 expressed separately demonstrates that their association can occur when proteins are combined after cell lysis. HEK293T cells were independently transfected with GP2-HA or CD163-FLAG plasmids. At 24 h post-transfection, cells were lysed and the lysates were mixed 1 : 1 and incubated with anti-FLAG beads. Negative controls included GP2-HA lysates mixed with empty-vector lysates. Inputs and immunoprecipitates were analysed by WB using anti-HA and anti-FLAG antibodies. (**c**) Replacement of the GP2 SP with that of EAV does not affect its interaction with CD163. SP sequences of PRRSV GP2 and EAV GP2 are shown above. The EAV GP2 SP was predicted using SignalP (d-score=0.745; cutoff=0.45). HEK293T cells were co-transfected with plasmids expressing the SP-EAV–GP2-HA mutant and CD163-FLAG. At 24 h post-transfection, lysates were analysed by WB (Input) and subjected to IP using anti-FLAG agarose beads. Negative controls included cells co-transfected with GP2-HA variants and empty pCDNA3.1. FLAG-peptide eluates were analysed by WB with anti-HA and anti-FLAG antibodies (IP). Right panels: Colocalization of SP-EAV–GP2-HA and CD163. HEK293T cells were transfected as described above and fixed at 24 h post-transfection for immunostaining with anti-FLAG (green) and anti-HA (red); nuclei were counterstained with DAPI (blue). Representative merged images showing colocalization (yellow) are shown.

Previous studies have shown that the viral envelope protein GP2 specifically interacts with the PRRSV entry receptor CD163 [16,27, 28]. To determine whether the GP2 SP influences this interaction, we examined the ability of the different SP–GP2 variants containing a C-terminal HA tag to associate with CD163, which was expressed with a C-terminal FLAG tag. To this end, we co-transfected similar amounts of plasmids expressing the GP2 variants together with the CD163 expression plasmid. Cells were lysed, and FLAG-tagged CD163 was immunoprecipitated using anti-FLAG beads. Eluted proteins were analysed by SDS–PAGE and WB with anti-HA and anti-FLAG antibodies (Fig. 2a). The results showed that removal of the SP from GP2 markedly reduced its ability to associate with CD163, suggesting that the SP contributes to efficient GP2–CD163 interaction. In contrast, preventing SP cleavage did not impair the ability of CD163 to associate with the GP2 variant (Fig. 2a). Quantification using a co-IP index (defined as the intensity of HA-tagged protein in the pellet normalized to input and CD163 levels) showed that deletion of the SP reduced GP2 association with CD163 to background levels, whereas mutations preventing SP cleavage had no significant effect (Fig. 2a). Colocalization experiments in co-transfected cells further confirmed the association of CD163 with SP-GP2* but not with ΔSP-GP2 (Fig. 2a). Together, these findings indicate that deletion of the GP2 SP disrupts GP2–CD163 association. However, given that SP removal also impairs ER localization, glycosylation and proper folding in the ER, this effect likely reflects broader defects in GP2 folding, maturation and trafficking rather than a specific or direct role of the SP in receptor engagement.

In a previous study, using co-IP and colocalization experiments, we showed that in transfected cells, CD163 interacts with GP2 in the ER, suggesting that the receptor and the viral envelope protein associate early after their biosynthesis [28]. To demonstrate that GP2–CD163 association does not require ER colocalization – and because the biologically relevant interaction between CD163 and GP2 is expected to occur extracellularly – we next examined the biochemical ability of FLAG-tagged CD163 to interact with HA-tagged GP2 under conditions in which the proteins were expressed separately. For this purpose, cells were independently transfected with plasmids expressing either CD163 or GP2. Cells were lysed, and the resulting lysates containing FLAG-tagged CD163 or GP2-HA were mixed. Following IP with anti-FLAG beads, proteins eluted with FLAG peptide were resolved by SDS–PAGE and analysed by WB using anti-FLAG and anti-HA antibodies. The co-IP assays showed that the two proteins were able to associate even when expressed separately and combined only after lysis (Fig. 2b). This indicates that their association does not strictly require co-translation and can occur under detergent-solubilized conditions, reflecting a reproducible biochemical association between full-length GP2 and CD163, potentially involving their ectodomain regions. Notably, we consistently observed stronger GP2–CD163 association when both proteins were co-expressed in the same cells compared to when lysates from separately transfected cells were mixed prior to IP (Fig. 2b). This suggests that co-expression facilitates more efficient complex formation. One possible explanation is that co-expression allows GP2 and CD163 to encounter each other within the same intracellular compartments during biosynthesis and trafficking, which may promote proper folding and post-translational maturation required for efficient association. In contrast, proteins expressed in separate cells are only combined after lysis, where differences in maturation state, loss of membrane context, or limited post-lysis interaction time may reduce the efficiency of association.

Next, we asked whether the replacement of the GP2 SP with the SP from EAV (SP-EAV-GP2) affected the ability of GP2 to interact with CD163. Compared with the PRRSV GP2 SP, the EAV GP2 SP is shorter and contains a more compact hydrophobic core with fewer polar residues, whereas the PRRSV SP is longer and enriched in additional N-terminal and C-terminal residues (Fig. 2c). Although the two SPs share little sequence homology, replacement of the GP2 SP with that of EAV generated a GP2 variant that localized to the ER but not to the Golgi and remained fully sensitive to Endo H digestion (Fig. S1A B), similar to WT GP2. The complete Endo H sensitivity and absence of Endo H–resistant forms further indicate that SP-EAV-GP2 does not reach the Golgi and is not processed into complex-type *N*-glycans. Thus, replacement of the GP2 SP with the EAV SP does not alter the intracellular trafficking of GP2. We next performed co-IP in cells co-expressing SP-EAV-GP2 and CD163, and the results showed that SP replacement did not impair the ability of CD163 to bind GP2 (Fig. 2c). Colocalization experiments in co-transfected cells further confirmed the association of CD163 with SP-EAV-GP2 (Fig. 2c). These findings confirm that the native GP2 SP is not required for GP2–CD163 association.

### Role of the GP2 SP in mediating interactions with other viral glycoproteins

**Fig. 3.**
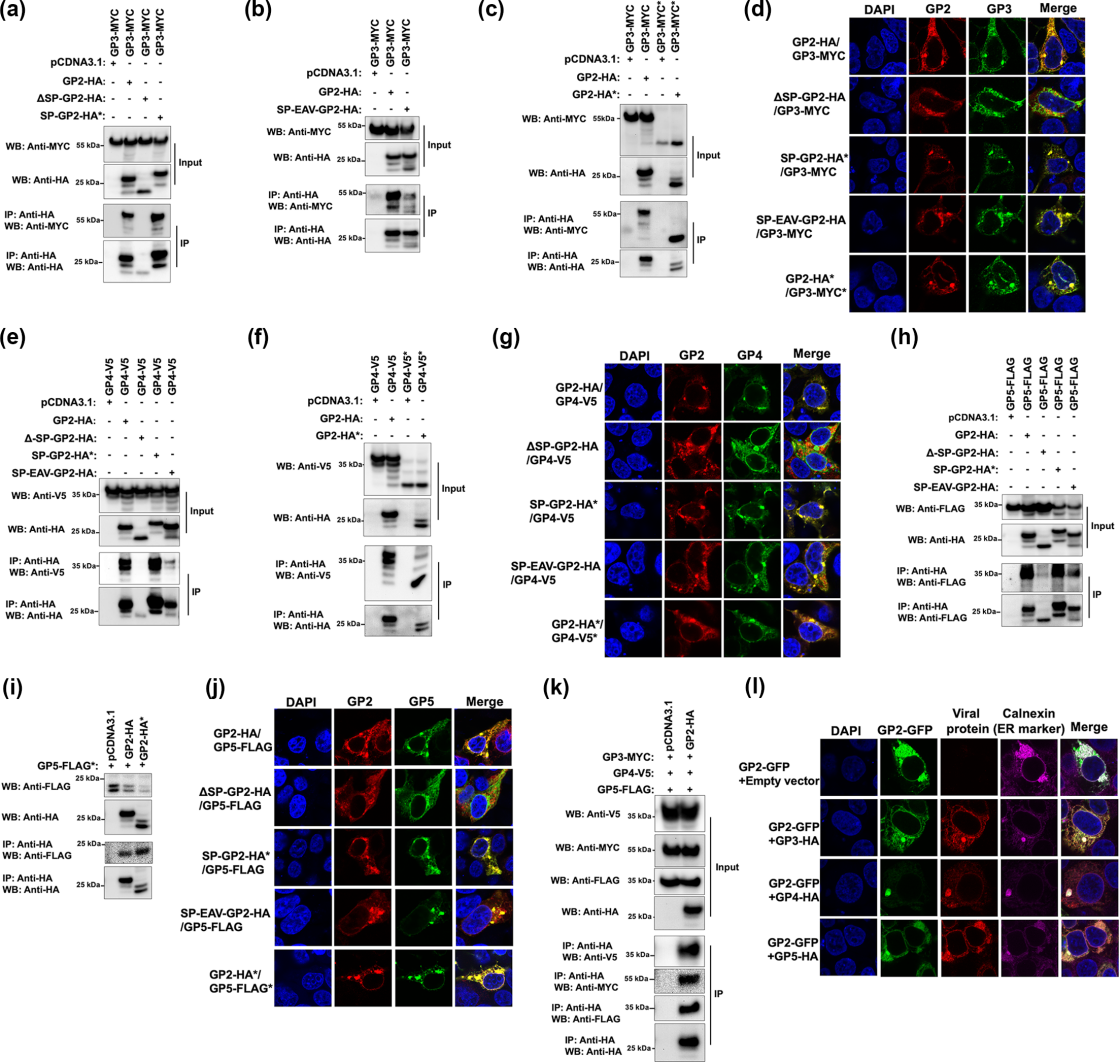
Effect of the GP2 SP on interaction with other viral glycoproteins. (**a**) Co-IP of GP2 variants with GP3. HEK293T cells were co-transfected with plasmids expressing WT or mutant GP2-HA and GP3-MYC. At 24 h post-transfection, lysates were analysed by WB with anti-HA and anti-MYC antibodies (Input) and subjected to IP using anti-HA agarose beads. To control for background binding, cells were co-transfected with GP3-MYC and an empty pCDNA3.1 vector. HA-peptide eluates were analysed by WB with anti-HA and anti-MYC antibodies (IP). (**b**) Replacement of the GP2 SP with that of EAV does not disrupt interaction with GP3. Same as in (**a**) but using EAV-SP–GP2-HA. (**c**) Blocking *N*-glycosylation of GP2 and GP3 does not disrupt their association. HEK293T cells were co-transfected with HA-tagged GP2 and MYC-tagged GP3 or their *N*-glycosylation-deficient variants (GP2-HA* and GP3-MYC*). Lysates were analysed and immunoprecipitated as in (**a**), with appropriate controls for nonspecific binding. (**d**) Colocalization of GP2 variants with GP3. Cells were transfected as indicated, fixed at 24 h and immunostained with anti-MYC (green) and anti-HA (red) antibodies; nuclei were counterstained with DAPI (blue). Representative merged images show colocalization (yellow). (**e**) Co-IP of GP2 variants with GP4. Same as in (**a**) and (**b**) but using a V5-tagged GP4 construct. (**f**) Blocking *N*-glycosylation of GP2 and GP4 does not disrupt their association. Same as in (**c**) but using a *N*-glycosylation-deficient GP4 variant (GP4-V5*). (**g**) Colocalization of GP2 variants with GP4. Same as in (**d**) but using V5-tagged GP4 constructs. Cells were immunostained with anti-V5 (green) and anti-HA (red) antibodies. (**h**) Co-IP of GP2 variants with GP5. Same as in (**a**) and (**b**) but using a FLAG-tagged GP5 construct. (**i**) Blocking *N*-glycosylation of GP2 and GP5 does not disrupt their association. Same as in (**c**) but using an *N*-glycosylation-deficient GP5 variant (GP5-FLAG*). (**j**) Colocalization of GP2 variants with GP5. Same as in (**d**) but using FLAG-tagged GP5 constructs. Cells were immunostained with anti-FLAG (green) and anti-HA (red) antibodies. (**k**) GP2 co-immunoprecipitates GP3, GP4 and GP5 when all four glycoproteins are co-expressed. Same as in (**a**) but cells were co-transfected with plasmids expressing GP2-HA, GP3-MYC, GP4-V5 and GP5-FLAG. (**l**) GP2 colocalizes with other viral glycoproteins in the ER. HEK293T cells were co-transfected with HA-tagged viral glycoproteins and GP2-GFP plasmids. After 24 h, cells were fixed and immunostained with anti-HA antibody (red). ER was visualized by staining cells with calnexin antibody (purple). GFP fluorescence was visualized directly (green). Nuclei were counterstained with DAPI (blue). Representative merged images show colocalization (yellow).

A previous report showed that the viral envelope protein GP2 interacts with the other viral envelope glycoproteins GP3, GP4 and GP5 [27]. To determine whether the GP2 SP influences these interactions, we first examined the ability of different SP–GP2 variants containing a C-terminal HA tag to bind GP3-MYC. To this end, we co-transfected cells with similar amounts of plasmids expressing the GP2 variants together with the GP3-MYC expression plasmid. Cells were lysed, HA-tagged GP2 variants were immunoprecipitated using anti-HA beads, and the eluted proteins were analysed by SDS–PAGE and WB with anti-HA and anti-MYC antibodies (Fig. 3a). The results showed that removal of the SP from GP2 markedly reduced its ability to bind GP3, indicating that the SP is required for efficient GP2–GP3 interaction (Fig. 3a). In contrast, preventing SP cleavage did not impair the ability of GP3 to bind the GP2 variant (Fig. 3a). Colocalization experiments in co-transfected cells further confirmed the association of GP3 with SP-GP2* but not with ΔSP-GP2 (Fig. 3d). Together, these findings indicate that deletion of the GP2 SP disrupts GP2’s ability to interact with GP3, likely because the SP is required for proper folding and maturation of GP2 in the ER, which in turn enables its interaction with GP3. Furthermore, co-IP results showed that replacement of the GP2 SP with the EAV SP did not affect the ability of GP2 to associate with GP3 (Fig. 3b), suggesting that the native GP2 SP is not specifically required for GP2–GP3 association. Colocalization experiments also confirmed this binding (Fig. 3d). Importantly, co-IP experiments also showed that removal of the *N*-glycosylation sites from either GP2 or GP3 did not affect their interaction, indicating that *N*-glycosylation is not required for the binding between these two glycoproteins (Fig. 3c). Colocalization experiments in co-transfected cells further confirmed the association of the two *N*-glycosylation-deficient mutants (Fig. 3d).

Next, we examined the role of the GP2 SP in its interaction with GP4 using V5-tagged GP4. Removal of the SP strongly reduced GP2–GP4 binding (Fig. 3e), indicating that the SP is required for efficient interaction. In contrast, preventing SP cleavage did not impair binding (Fig. 3e). Colocalization in co-transfected cells confirmed that GP4 associates with SP-GP2* but not with ΔSP-GP2 (Fig. 3g). Replacement of the GP2 SP with that of EAV did not affect GP2–GP4 association, as shown by both co-IP (Fig. 3e) and colocalization (Fig. 3g), indicating that the native PRRSV SP is not specifically required. Finally, removal of *N*-glycosylation sites from either GP2 or GP4 did not disrupt their interaction (Fig. 3f), and colocalization of the corresponding mutants further supported this result (Fig. 3g).

Our next step was to test whether the GP2 SP influences its interaction with GP5 using FLAG-tagged GP5. Removing the SP sharply reduced GP2–GP5 binding, indicating that the SP is required for efficient interaction (Fig. 3h). In contrast, blocking SP cleavage did not impair binding. Colocalization confirmed that GP5 associates with SP-GP2* but not with ΔSP-GP2 (Fig. 3j). Replacing the GP2 SP with the EAV SP did not affect GP2–GP5 binding, as shown by both Co-IP and colocalization (Fig. 3h, j), indicating that the native PRRSV SP is not specifically required. Finally, removing *N*-glycosylation sites from either GP2 or GP5 did not disrupt their interaction (Fig. 3i), a result further supported by colocalization of the *N*-glycosylation-deficient mutants (Fig. 3j). Overall, these results demonstrate that the GP2 SP is essential for GP2 to interact efficiently with GP3, GP4 and GP5, but the specific PRRSV SP sequence is not required, and *N*-glycosylation of either partner does not contribute significantly to these interactions.

To further determine whether GP2 can pull down the full multiprotein glycoprotein complex, we transiently co-expressed all four viral envelope glycoproteins, each carrying a distinct epitope tag and performed IP using anti-HA beads to isolate HA-tagged GP2. The results showed that GP3, GP4 and GP5 were all co-immunoprecipitated with GP2 (Fig. 3k), demonstrating that GP2 is capable of pulling down the entire glycoprotein complex simultaneously. This finding confirms that GP2 directly associates with the assembled minor-major glycoprotein complex.

Interestingly, our results indicated above showed that removing the GP2 SP disrupted its ability to associate with the other viral glycoproteins. Colocalization experiments similarly demonstrated that ΔSP-GP2 failed to colocalize with the viral glycoproteins and did not localize properly to the ER (Fig. 1d), suggesting that these interactions occur in the ER early after protein synthesis. To test this hypothesis, we examined the intracellular distribution of GP2-EGFP co-expressed with each viral glycoprotein. As shown in Fig. 3l, GP2-EGFP and the other envelope proteins colocalized with calnexin, confirming that GP2 and the viral glycoproteins interact shortly after their biogenesis and that their assembly occurs initially in the ER.

### Role of the GP2 SP in PRRSV-2 infection

**Fig. 4.**
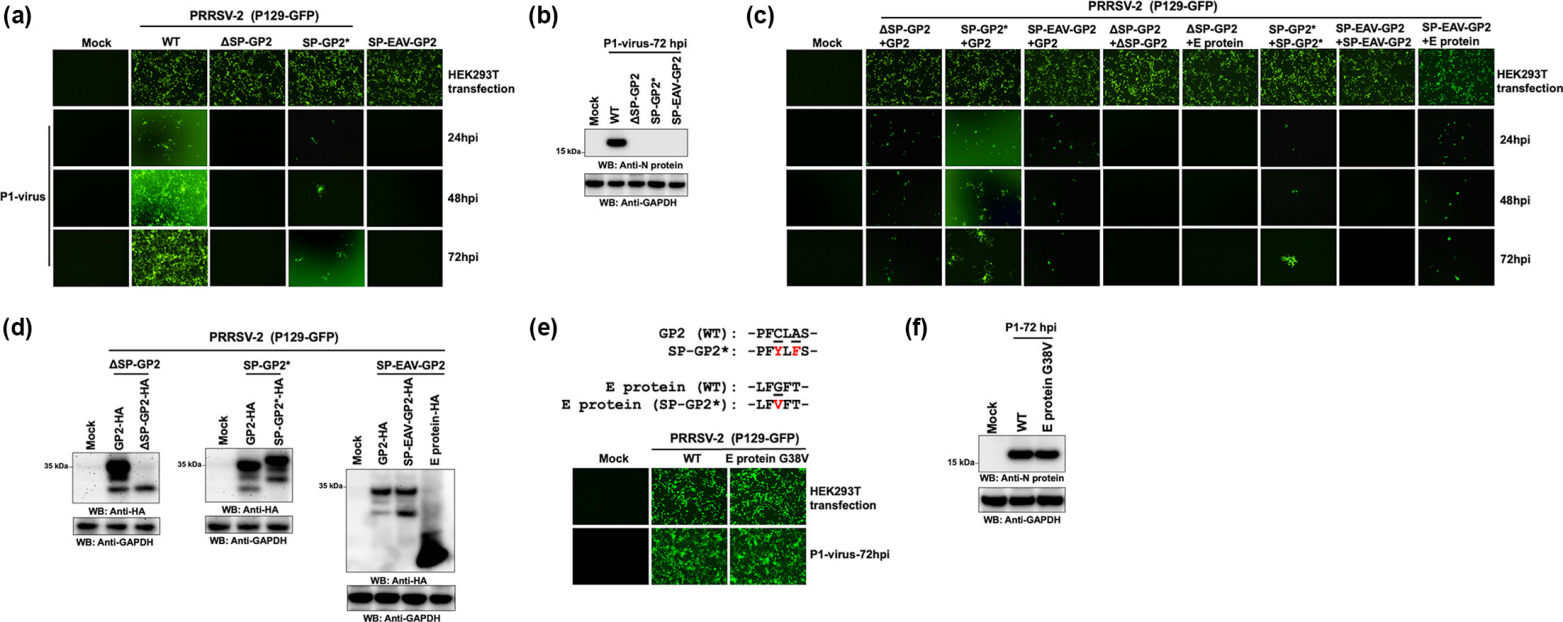
Effect of the GP2 SP on PRRSV-2 infection. (**a**) The GP2 SP is required for viral infection. Supernatants from HEK293T cells transfected with the indicated PRRSV-2 cDNA clones expressing GFP (P129-GFP) were used to infect MARC-145 cells. Infected cells were fixed at the indicated hours post-infection (hpi) and visualized under fluorescence microscopy. (**b**) Supernatants from HEK293T cells transfected with the indicated PRRSV-2 cDNA clones were used to infect MARC-145 cells. At 72 hpi, cells were lysed and analysed by SDS–PAGE and WB with an anti-N monoclonal antibody. GAPDH was used as a loading control. (**c**) Trans-complementation of non-infectious clones. HEK293T cells were co-transfected with the indicated PRRSV-2 cDNA clones expressing GFP (P129-GFP) and GP2 variants containing a C-terminal HA tag. Culture supernatants were harvested at 72 h post-transfection (hpt) and used to inoculate MARC-145 cells. Infected cells were fixed at the indicated hpi and visualized under fluorescence microscopy. (**d**) Expression analysis of GP2 variants containing a C-terminal HA tag co-expressed with PRRSV-2 cDNA clones. HEK293T cells were co-transfected with the indicated PRRSV-2 cDNA clones expressing GFP (P129-GFP) and GP2 variants containing a C-terminal HA tag. At 72 hpt, cells were lysed and analysed by SDS–PAGE and WB with an anti-HA monoclonal antibody. GAPDH was used as a loading control. Mock: cells transfected with an empty vector. (**e**) Mutations in GP2 that prevent SP cleavage alter the overlapping E protein sequence but do not abolish infection. Amino acid sequences of WT GP2 and the SP–GP2* mutant around the SP cleavage site, as well as the resulting sequences of the E protein, are shown (top). The −1 and −3 residues, which were mutated to prevent cleavage by the signal peptidase, are underlined, along with the amino acid change introduced in the E protein. Mutated residues are shown in red. Supernatants from HEK293T cells transfected with the indicated PRRSV-2 cDNA clones expressing GFP (P129-GFP) were used to infect MARC-145 cells. Infected cells were fixed at 72 hpi and visualized under fluorescence microscopy. (**f**) Supernatants from HEK293T cells transfected with the indicated PRRSV-2 cDNA clones were used to infect MARC-145 cells. At 72 hpi, cells were lysed and analysed by SDS–PAGE and WB with an anti-N monoclonal antibody. GAPDH was used as a loading control.

To analyse the effect of the GP2 SP on PRRSV infection, we generated infectious GFP-expressing clones (P129-GFP) carrying the same GP2 mutations described in the previous sections, which were used to map the SP cleavage site. The modified clones were transfected into HEK-293T cells, and 48 h later, the culture supernatants were used to inoculate MARC-145 cells. As expected, the WT clone efficiently infected MARC-145 cells, as evidenced by robust GFP expression and *N*-protein detection by WB (Fig. 4a, b). Removal of the GP2 SP (ΔSP-GP2) produced a clone that failed to infect MARC-145 cells, since no GFP-positive cells or *N*-protein expression were detected (Fig. 4a, b). This result is consistent with our earlier findings showing that SP deletion disrupts GP2’s ability to associate with the other viral glycoproteins (Fig. 3). Preventing SP cleavage (SP-GP2*) also impaired infection, indicating that SP cleavage is required for productive replication. Although a few scattered GFP-positive cells were observed (Fig. 4a), they were insufficient to support sustained viral replication, as no *N*-protein was detected (Fig. 4b). Likewise, replacing the GP2 SP with that of EAV (SP-EAV-GP2) abolished infectivity (Fig. 4a, b), indicating that the PRRSV GP2 SP cannot be functionally substituted.

Because the GP2 coding sequence overlaps with the E-protein reading frame, mutations introduced into the GP2 SP also disrupt E-protein expression, which is essential for PRRSV infection [49]. Thus, the observed loss of infectivity may result from impaired E-protein synthesis rather than from GP2 SP defects alone. We, therefore, tested whether infectivity could be restored by supplying GP2 or E in trans. To determine whether GP2 expression in trans rescues virus production, HEK-293T cells were co-transfected with the mutant clones and a plasmid expressing GP2-HA. As negative controls, the mutant clones were co-transfected with GP2-HA variants containing the same SP mutations. WB using anti-HA antibodies confirmed co-expression of the GP2 constructs with the infectious clones (Fig. 4d). Supernatants collected 48 h post-transfection were transferred onto MARC-145 cells, and infection was assessed by fluorescence microscopy. MARC-145 cells inoculated with supernatants from cells co-transfected with WT GP2-HA exhibited GFP expression, indicating that replication of the mutant clones can be rescued by GP2 supplied in trans (Fig. 4c). In contrast, co-expression with the corresponding GP2 SP mutants failed to restore infectivity (Fig. 4c), demonstrating that the infectivity defects arise specifically from the GP2 SP mutations. To further distinguish the effects of GP2 SP mutations from potential disruption of the overlapping E gene, we performed rescue experiments in which the E protein was supplied in trans. The ΔSP-GP2 clone was not rescued by E-protein expression, indicating that its loss of infectivity results from removal of the SP from GP2 rather than from impaired E-protein synthesis (Fig. 4c). In contrast, infectivity of the SP-EAV-GP2 clone was rescued by E-protein expression, indicating that the loss of infectivity in this mutant was primarily due to disrupted E-protein synthesis caused by SP replacement (Fig. 4c). This result also suggests that the GP2 SP can be functionally substituted, provided that E-protein expression is maintained.

Sequence analysis of the SP-GP2* clone revealed that the SP-cleavage-blocking mutation alters the overlapping E-protein ORF, substituting glycine 38 with valine (G38V) (Fig. 4e). To confirm that this mutation does not account for the loss of infectivity, we generated a clone containing only the G38V mutation and tested its infectivity as described above. The G38V mutant infected MARC-145 cells comparably to the WT clone (Fig. 4e, f), demonstrating that the G38V mutation does not impair infection and that the loss of infectivity in SP-GP2* results from the prevention of SP cleavage. Because of the overlapping reading frames, introduction of the G38V substitution in the E protein also results in an alanine-to-serine substitution at position 40 of GP2. However, this substitution is not predicted to affect SP cleavage, as indicated by a SignalP 3.0 d-score of 0.838 and by Phobius and PolyPhobius predictions. This is consistent with the fact that both alanine and serine are small, helix-compatible residues typically tolerated at the −1 and −3 positions required for efficient signal peptidase recognition and cleavage [46,48]. Consistent with this prediction, WB analysis comparing the migration patterns of WT GP2-HA, SP-GP2-HA* and a GP2-HA variant carrying the A40S substitution showed that the A40S mutant migrates similarly to WT GP2-HA and distinctly from SP-GP2-HA*, confirming that this substitution does not interfere with SP cleavage (data not shown).

### Identification of GP2 regions required for interaction with CD163

**Fig. 5.**
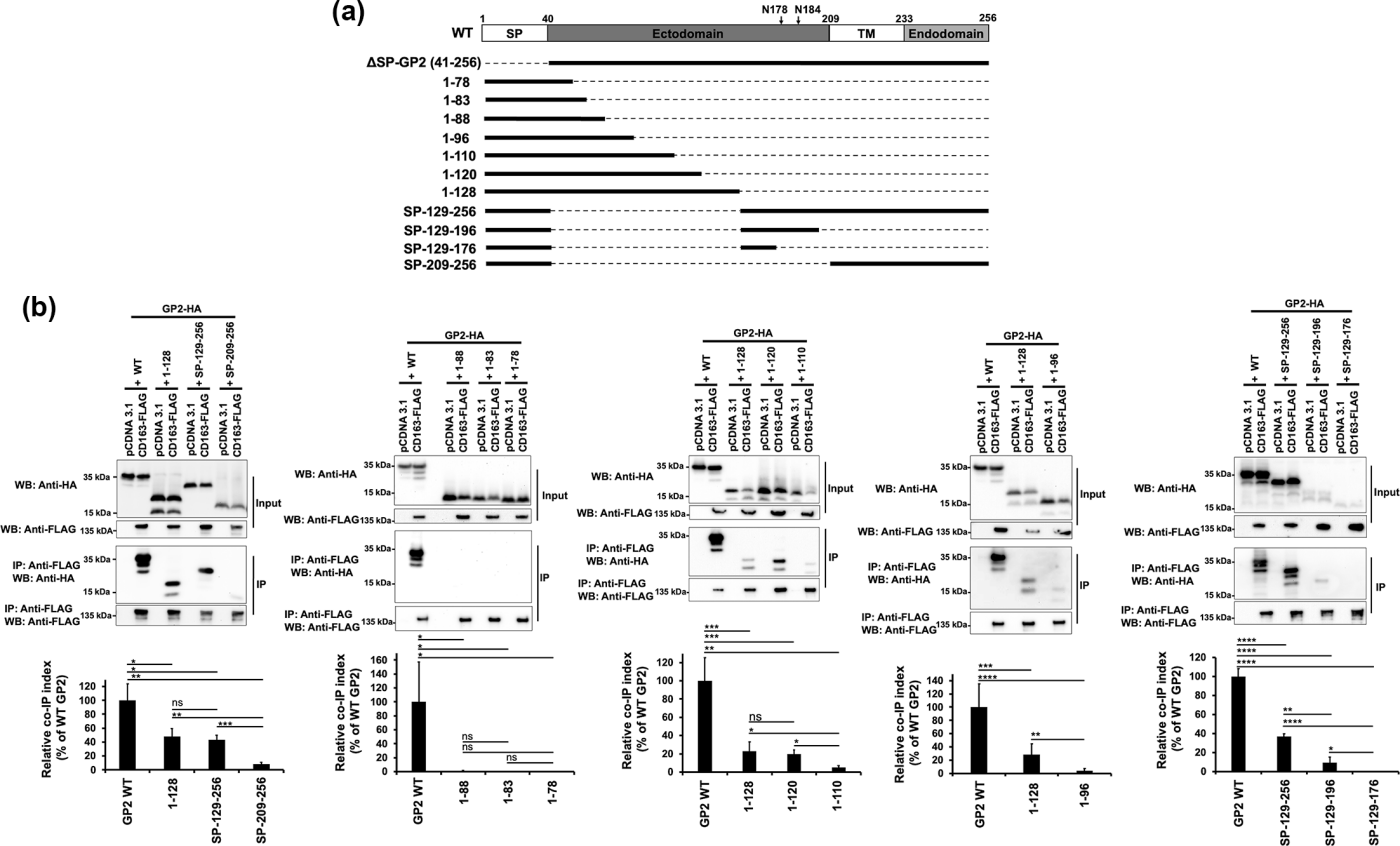
CD163 receptor–binding capacity of GP2 mutants. (**a**) The WT PRRSV-2 GP2 glycoprotein is depicted at the top. The amino acid residue numbers at the boundaries of the GP2 domains are indicated. GP2 variants are shown below, with dashed lines representing deleted sequences. (**b**) Co-IP analysis of CD163 and GP2 deletion mutants. HEK293T cells were co-transfected with plasmids expressing WT or mutant GP2-HA and CD163-FLAG. At 24 h post-transfection (hpt), cell lysates were analysed by WB with anti-HA and anti-FLAG antibodies (Input) and subjected to iIP using anti-FLAG agarose beads. To control for background binding, cells were co-transfected with GP2-HA and an empty pCDNA3.1 vector. FLAG-peptide eluates were analysed by WB with anti-HA and anti-FLAG antibodies (IP). The intensity of the WB bands was quantified using ImageJ software. The strength of the interaction between CD163 and the different GP2 variants was evaluated, and a co-IP index was calculated for each co-IP reaction as described in Methods (*P<0.05; **P<0.01; ***P<0.001; ****P<0.0001; ns, not significant, *P*≥0.05).

To define the regions of GP2 required for interaction with the viral receptor CD163, we generated a series of GP2 deletion mutants to map the minimal domains necessary for GP2–CD163 association (Fig. 5a). Our previous results showed that removal of the GP2 SP abolishes GP2–CD163 binding, most likely because proper ER targeting and folding are required for this interaction. Therefore, to specifically examine CD163-interacting regions within the GP2 ectodomain, we designed two complementary sets of deletion constructs: (i) mutants lacking different portions of the C-terminal SP/ectodomain and (ii) constructs in which the second half of GP2 [including the remaining ectodomain, transmembrane (TM) region and cytoplasmic tail] was fused to the SP to preserve ER targeting and allow proper folding. This strategy enabled us to assess CD163-binding capability while maintaining the structural context needed for GP2 maturation.

To evaluate the association between CD163 and each GP2 variant, we performed co-IP assays using HA-tagged GP2 mutants and CD163-FLAG co-expressed in transfected cells. Compared with WT GP2, the mutant containing only the first 128 residues (1–128) displayed reduced association with CD163, indicating that this region includes – but does not fully encompass – a determinant required for efficient CD163 binding (Fig. 5b and Table 1). Consistent with this, the complementary construct in which the SP was fused to residues 129–256 (SP-129–256) also interacted with CD163, suggesting that an additional CD163-interacting determinant resides within this region. In contrast, the construct containing the SP fused solely to the TM and cytoplasmic domains co-immunoprecipitated only minimal amounts of CD163, demonstrating that these regions contribute little to GP2–CD163 association. Quantification using a co-IP index (defined as the intensity of HA-tagged protein in the pellet divided by the sum of HA-tagged protein in the input and FLAG-tagged CD163 in the pellet) supported these conclusions (Fig. 5b). Together, these data indicate that CD163-interacting determinants are confined to the GP2 ectodomain, whereas the TM and cytoplasmic tail are dispensable for CD163 binding.

**Table 1.** Phenotypes of GP2 deletion variants

GP2 variant	Association with CD163*	Colocalization†
**WT**	**+++**	**+**
**41–256**	−	−
**1–78**	−	−
**1–83**	−	−
**1–88**	−	−
**1–96**	**+**	**+**
**1–110**	**+**	**+**
**1–120**	**++**	**+**
**1–128**	**++**	**+**
**SP-129-256**	**++**	**+**
**SP-129-196**	**+**	**+**
**SP-129-176**	−	−
**SP-209-256**	**+**	−

*aGP2 variants were assayed for their ability to interact with CD163-FLAG, as described in Methods. ‘+++’ indicates 100% association, corresponding to the amount of WT GP2 that co-immunoprecipitates with CD163-FLAG; ‘++’ indicates ≈45-20% association; ‘+’ indicates <20% association; and ‘−’ indicates no detectable interaction. Similar results were obtained in three independent experiments.

†Colocalization of GP2 variants with CD163 was determined as described in Methods. ‘+’ indicates colocalization; ‘−’ indicates no colocalization. Experiments were performed at least three times, and representative results are shown.

WT, wild type.

To identify the minimal region within the first 128 residues that mediates CD163 binding, we generated a panel of additional deletion mutants (Fig. 5a) and performed co-IP assays as described above. Constructs containing residues 1–128 and 1–120 exhibited comparable CD163 binding, indicating that the final eight residues of the 1–128 fragment are dispensable (Fig. 5b and Table 1). In contrast, mutants 1–110 and 1–96 showed a significant reduction in binding relative to 1–128, and constructs 1–78, 1–83 and 1–88 were completely unable to associate with CD163 (Fig. 5b and Table 1). These patterns support the presence of a critical CD163-interacting determinant within residues 88–120. Moreover, the decreased binding observed when residues just downstream of the 88–110 core were removed suggests that the functional binding region spans the entire 88–120 interval rather than being restricted to a narrower 96–110 segment (Fig. 5b and Table 1).

**Fig. 6.**
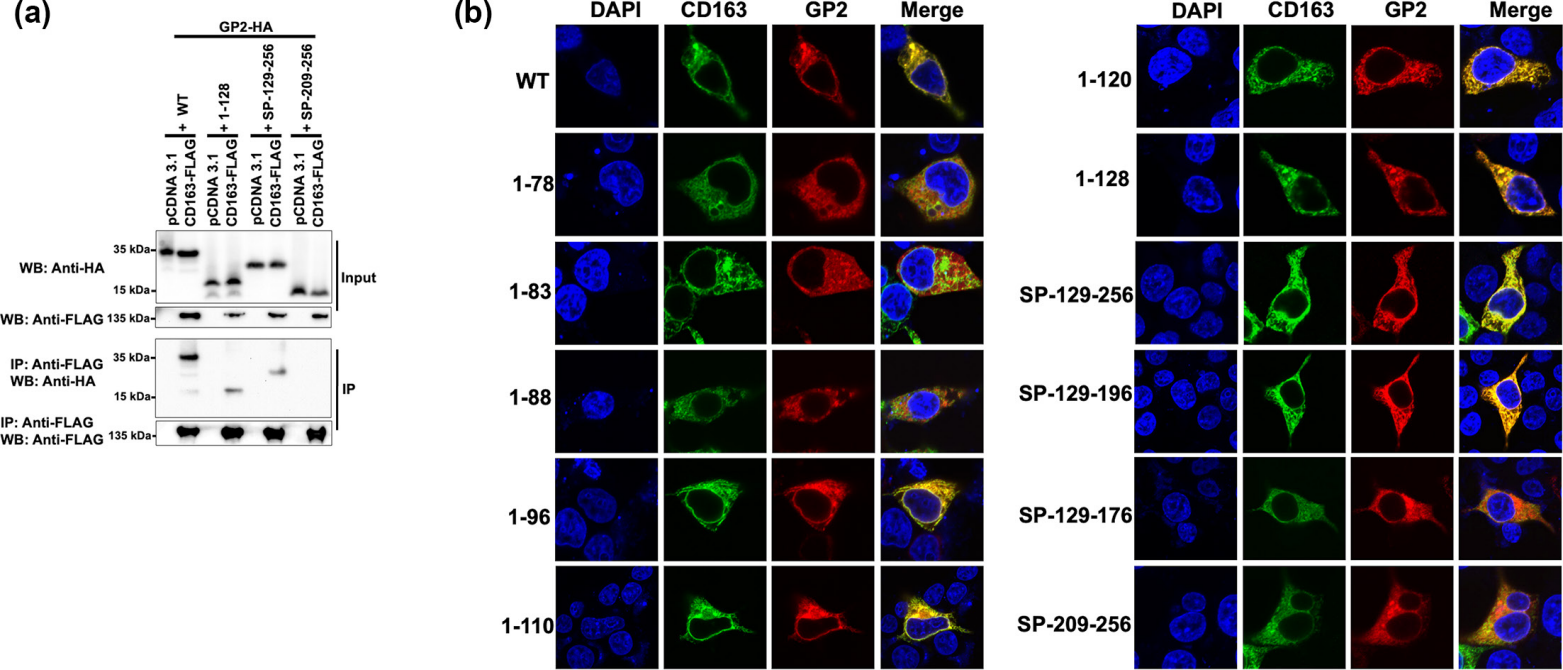
Co-IP of GP2 variants and CD163 expressed separately indicates a stable association. (**a**) HEK293T cells were independently transfected with plasmids expressing GP2-HA variants or CD163-FLAG. At 24 hpt, cells were lysed, and equal volumes of the two lysates were mixed (1 : 1) and incubated with anti-FLAG agarose beads. Negative controls included GP2-HA lysates mixed with lysates from cells transfected with an empty vector. Input and immunoprecipitated fractions were analysed by WB using anti-HA and anti-FLAG antibodies. (**b**) Colocalization of GP2 variants with CD163. Cells were transfected as indicated, fixed at 24 h and immunostained with anti-FLAG (green) and anti-HA (red) antibodies; nuclei were counterstained with DAPI (blue). Representative merged images show colocalization (yellow).

To define the second CD163-interacting region located within residues 129–256, we generated additional deletion mutants of this portion of GP2 (Fig. 5a). The results showed that the mutant SP-129–176 failed to bind CD163, whereas the construct containing the SP fused to a more distal ectodomain region (SP-129-196) retained the ability to interact (Fig. 5b and Table 1). These data suggest that the second interaction motif resides between residues 176 and 196. Notably, deletion of the TM region and cytoplasmic tail from the SP-129–256 construct markedly reduced CD163 binding, suggesting that these domains may contribute indirectly to receptor interaction by stabilizing ectodomain conformation or facilitating proper presentation of the CD163-interacting region. Similar roles for membrane-proximal and TM domains in maintaining structural integrity and functional receptor engagement have been described for other viral envelope glycoproteins [50]. To verify this – and because the physiologically relevant CD163–GP2 interaction occurs extracellularly – we next assessed the biochemical interaction between FLAG-tagged CD163 and HA-tagged GP2 variants expressed independently and combined only after cell lysis. Co-IP assays demonstrated that the two GP2 mutants containing the putative CD163-interacting regions (1–128 and SP-129–256) associated with CD163 even when expressed separately (Fig. 6a). In contrast, the SP-209–256 mutant, which contains only the TM region and cytoplasmic tail, did not bind CD163. Together, these results indicate that GP2 contains two distinct ectodomain regions – residues 88–120 and 176–196 – that are required for efficient association with CD163. Although the TM region and cytoplasmic tail are not directly involved in receptor binding, they appear to facilitate interaction of the second binding region with CD163, likely by contributing to proper folding or presentation of the ectodomain. Colocalization analyses in co-transfected cells further supported these findings, as GP2 constructs containing residues 88–120 or 176–196 associated with CD163, whereas variants that failed to co-precipitate with CD163 also did not colocalize with the receptor (Fig. 6b and Table 1).

### Deletion of either of the two GP2 ectodomain regions required for CD163 interaction markedly impaired infectivity in MARC-145 cells

**Fig. 7.**
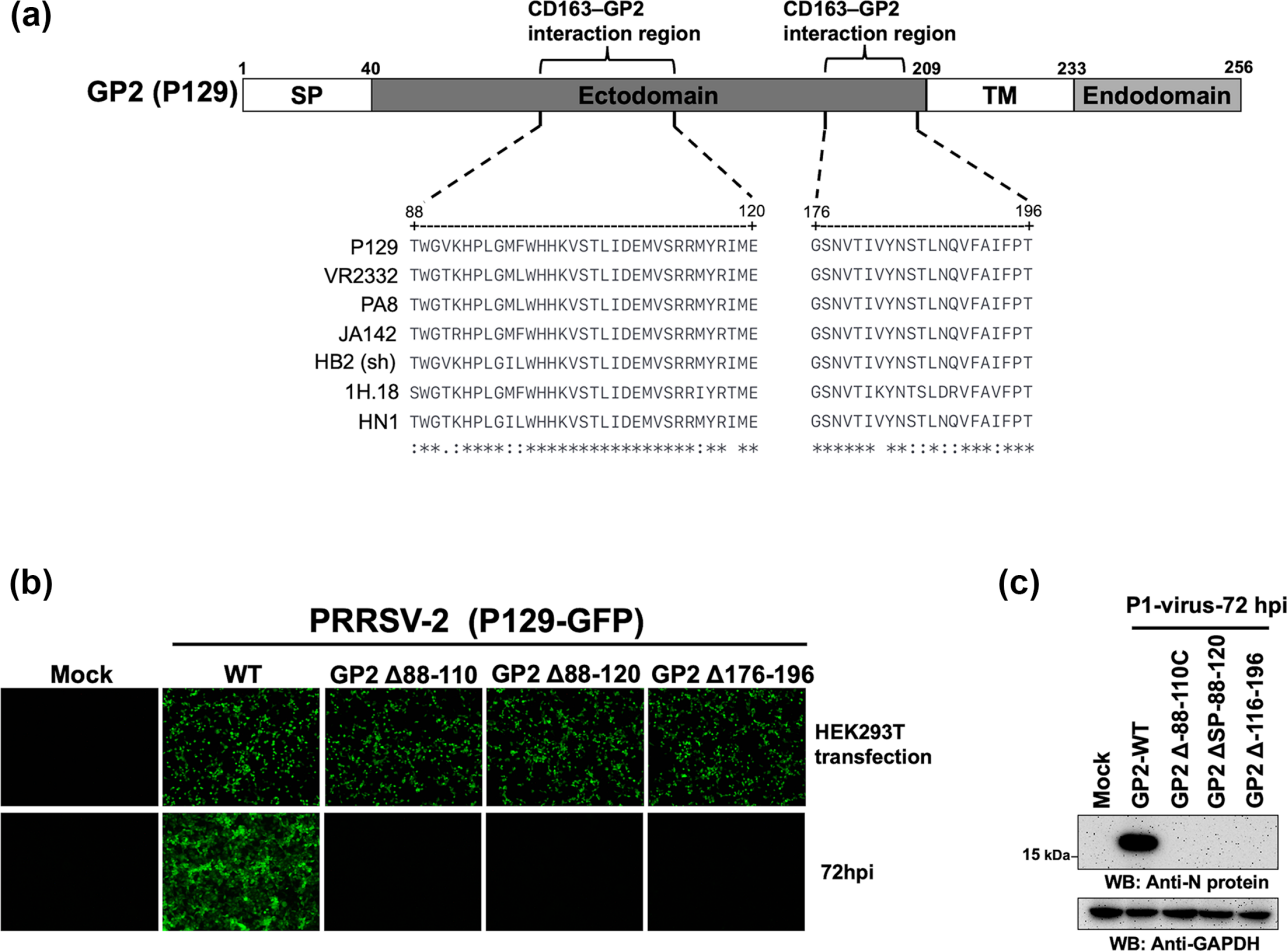
Deletion of either GP2 ectodomain region required for CD163 binding prevents viral infection. (**a**) Schematic representation of GP2 domains is shown at the top. The amino acid alignment below highlights conserved regions within the GP2 ectodomain corresponding to the mapped CD163-binding motifs. Sequences from representative PRRSV-2 isolates are shown, with conserved residues indicated by asterisks and conservative substitutions by dots. Alignments of GP2 amino-acid segments corresponding to residues 88–120 and 176–196 were generated using MAFFT v7 [82]. (**b**) The GP2 CD163-interacting domains are essential for viral infection. Supernatants from HEK-293T cells transfected with the indicated GFP-expressing PRRSV-2 cDNA clones (P129-GFP) were used to infect MARC-145 cells. Infected cells were fixed at 72 hpi and visualized by fluorescence microscopy. (**c**) Supernatants from HEK-293T cells transfected with the indicated PRRSV-2 cDNA clones were used to infect MARC-145 cells. At 72 hpi, cells were lysed and analysed by SDS–PAGE and WB using an anti-N monoclonal antibody. GAPDH was used as a loading control.

Sequence alignment of GP2 from seven representative PRRSV-2 isolates (P129, GenBank: QGD14182.1; VR2332, GenBank: AAD12126.1; PA8, GenBank: AAG13727.1; JA142, GenBank: AAR88265.1; HB-2(sh)/2002, GenBank: AAP57402.1; 1 h.18, GenBank: XLC13383.1; HN1, GenBank: AAR19402.1) showed that the two minimal CD163-binding domains (residues 88–120 and 176–196) are highly conserved, with 97 and 95% of positions, respectively, being either identical or conservatively substituted across strains (Fig. 7a). This high level of conservation indicates a strong functional constraint on the GP2–CD163 interaction interface.

To evaluate the effect of deleting these regions on PRRSV-2 infection, we generated infectious GFP-expressing clones (P129-GFP) carrying specific deletions within the conserved GP2 domains required for CD163 binding. The modified clones were transfected into HEK-293T cells, and 48 h later, culture supernatants were used to inoculate MARC-145 cells. As expected, the WT clone efficiently infected MARC-145 cells, as indicated by robust GFP expression (Fig. 7b) and N protein detection by WB (Fig. 7c). In contrast, deletion of either CD163-interacting region produced clones that failed to infect MARC-145 cells, as no GFP-positive cells were detected (Fig. 7b) and no N protein expression was observed (Fig. 7c). Even a smaller deletion (Δ88–110) within the first interacting domain markedly impaired the ability of the recombinant virus to infect permissive cells (Fig. 7b, c). These findings are fully consistent with our biochemical mapping data and demonstrate that both GP2 ectodomain motifs are essential for CD163-mediated viral infection.

## Discussion

In this study, we define the role of the GP2 SP in PRRSV-2 glycoprotein biogenesis, receptor engagement and viral infection, and we identify two highly conserved, discrete ectodomain regions of GP2 that are essential for interaction with the entry receptor CD163 and for productive infection. Altogether, these findings define critical determinants of GP2–CD163 specificity and significantly enhance our understanding of the molecular events that drive PRRSV entry. Our biochemical and cell biological experiments demonstrate that the N-terminal segment comprising the first 40 residues of GP2 functions as a canonical SP that is cleaved co- or post-translationally and is both necessary and sufficient for ER targeting. This is consistent with the established paradigm that canonical SPs mediate both ER targeting and post-targeting regulatory functions [51]. The combined use of SignalP, Phobius and PolyPhobius predictions, −1/−3 residue mutagenesis and comparative mobility analysis of WT GP2, ΔSP-GP2 and a signal-peptide–blocking mutant (SP-GP2*) provides direct experimental validation of a GP2 SP that had previously been inferred only from sequence analysis [22,42]. The complete Endo H sensitivity of GP2 and its robust colocalization with calnexin indicate that GP2 is retained in the early secretory pathway, consistent with the ER/ER–Golgi intermediate compartment (ERGIC) being a major site of arterivirus assembly [52]. The finding that fusion of the GP2 SP to GFP is sufficient to redirect GFP to the ER further underscores that this SP behaves as a classical targeting sequence. Functionally, the SP is indispensable for GP2 maturation and *N*-glycosylation. Deletion of the SP (ΔSP-GP2) abolishes ER localization, prevents *N*-glycan acquisition, and disrupts interactions with GP3, GP4, GP5 and CD163. In contrast, preventing SP cleavage (SP-GP2*) preserves ER targeting and allows detectable interactions with partner glycoproteins and CD163, yet is incompatible with virus replication. This dichotomy suggests that SP presence and SP cleavage contribute at distinct steps: (i) the SP itself is required for ER targeting and initial folding, whereas (ii) SP cleavage is required to generate a mature N-terminus competent for virion incorporation and entry. Notably, analogous observations have been made in herpesviruses: for Herpes Simplex Virus 1 (HSV-1), proper processing of the glycoprotein K (gK) SP by signal peptide peptidase (SPP) is essential for viral spread, and pharmacologic inhibition of gK–SPP interactions markedly reduces viral replication [53,56]. Since our results show that preventing GP2 SP cleavage abolishes PRRSV infection, it will be important to determine whether targeting GP2 SP processing may similarly represent a therapeutic strategy for inhibiting PRRSV entry.

An important implication of these findings is that GP2 folding, complex assembly and infectivity are highly sensitive to the architecture of its N-terminus. Although the EAV-derived SP can substitute for the native PRRSV SP to direct GP2 to the ER and support its interaction with GP3, GP4, GP5 and CD163, replacement of the endogenous SP eliminated infectivity in the context of the full-length viral genome unless E protein was supplied in trans. Because the GP2 SP overlaps the E open reading frame, these complementation experiments disentangle GP2-specific defects from those arising from altered E expression and demonstrate that SP mutations can simultaneously perturb minor-envelope protein stoichiometry and GP2 maturation. This is consistent with previous studies showing that, although the minor envelope proteins (GP2, GP3, GP4 and E) are dispensable for particle assembly, they form an essential multimeric entry complex required for infectivity [22] and that E likely functions as an ion channel facilitating early uncoating [49].

Our co-IP and colocalization data show that the GP2 SP is essential for efficient interaction with GP3, GP4 and GP5, whereas its precise primary sequence is not. Thus, correct ER targeting and early entry into the secretory pathway – rather than SP-specific amino acid motifs – appear to be the key requirements for assembly of the GP2–GP3–GP4/GP5 complex. The observation that GP2 can simultaneously pull down GP3, GP4 and GP5 supports the idea that GP2 may act as a central organizing component within a larger envelope glycoprotein assembly, consistent with previous findings showing that GP2 and GP4 interact with all other viral glycoproteins to form a multiprotein complex [27]. Notably, our experiments showed that *N*-glycosylation of GP2 or its partners is not required for their physical association, indicating that glycans primarily modulate folding and maturation rather than serving as determinants of the core glycoprotein–glycoprotein interface. This interpretation aligns with an earlier study reporting that removal of *N*-glycosylation sites from PRRSV viral envelope glycoproteins reduces their expression and biosynthetic efficiency but not their association with CD163 [26]. Similar observations have been reported for other viral systems, including Hepatitis B Virus envelope proteins [57,58], Hantaan Virus glycoproteins [59] and Ebola Virus glycoprotein [60]. In addition, our colocalization experiments revealed that GP2 associates with the other viral glycoproteins within the ER, suggesting that these interactions occur shortly after protein biogenesis and that assembly of the GP2–GP3–GP4 complex begins in the ER. This is consistent with recent work showing that PRRSV assembly occurs in the endoplasmic reticulum (ER) and the ERGIC [52].

The importance of GP2 in viral infection has been previously demonstrated, as viruses lacking ORF2a are unable to replicate in MARC-145 cells and PAMs [61]. Although GP2 is not required for the production of virus-like particles, these particles are non-infectious, indicating that viral entry is impaired in its absence [22]. Previous reports have demonstrated that GP2, together with other viral glycoproteins (GP3, GP4 and GP5), associates with CD163 and contributes to PRRSV entry, including processes involved in viral uncoating and genome release from early endosomes [12,16, 28, 29, 62, 63]. Specifically, GP2 has been reported to associate with CD163 [16,27, 28]. Notably, deletion of the SRCR5 domain from CD163 in a modified MARC-145 cell line did not affect the colocalization of CD163 with PRRSV in early endosomes, but it reduced interactions between CD163 and GP2 (and other entry-associated viral glycoproteins), which are required for virus uncoating, resulting in a block in productive infection [16]. Together, these findings suggest that domain 5 of CD163 is a key determinant of CD163-mediated viral entry, including the GP2–CD163 association during PRRSV infection. However, a more recent study showed that deletion of CD163 regions required for viral infection did not abolish its association with GP2 and other viral glycoproteins, supporting a ‘multi-domain’ model in which PRRSV glycoproteins engage multiple regions of CD163 [12,29].

Recent studies have identified multiple residues within GP2a as determinants of PRRSV tropism. Residue 118 influences viral attachment to PAMs and modulates association with CD163, particularly involving the SRCR5 domain [64]. Similarly, residue 160 contributes to macrophage infectivity and *in vivo* virulence, as the K160I substitution reduces PAM infection and attenuates disease while maintaining CD163 dependence [34]. In addition, residues 91, 97 and 98 play key roles in viral adaptation to MARC-145 cells, with substitutions, such as F98L enhancing replication *in vitro* without significantly affecting pathogenicity. These effects are strain-dependent and may also influence infectivity in PAMs and piglets [32,33].

A central advance of this work is the mapping of two distinct regions in the GP2 ectodomain required for association with CD163, spanning residues 88–120 and 176–196. Both regions reside in highly conserved portions of GP2 among PRRSV-2 isolates, and deletion of either segment abolishes infection, demonstrating that they are essential for CD163-mediated entry rather than simply contributing to association efficiency. Notably, while these experiments were performed in a transfection system in the absence of other viral proteins, our colocalization and co-IP assays do not distinguish between direct and indirect interactions. Therefore, we cannot conclude that GP2 directly binds CD163, and it remains possible that cellular factors contribute to or stabilize the observed association. Future studies using purified proteins or biophysical binding assays will be required to determine whether a direct interaction occurs.

Previous studies localized CD163 dependence to the scavenger receptor cysteine-rich (SRCR) domain 5 and showed that GP2 and GP4 can interact with CD163 and participate in genome release from early endosomes [12,16, 28, 29, 62, 63]. Our findings extend this model by defining the viral side of the interface and revealing that CD163 recognizes at least two spatially distinct sites within the GP2 ectodomain. This interpretation is further supported by biochemical assays in which FLAG-tagged CD163 and HA-tagged GP2 variants, expressed independently and mixed only after cell lysis, associated exclusively when the GP2 variant contained one of the two CD163-interacting domains. The use of multiple receptor-interacting domains within a single viral envelope glycoprotein has been described for other viruses. For example, Feline Leukaemia Virus subgroup C engages its receptor FLVCR1 through two distinct domains: an N-terminal receptor-binding domain and a C-terminal domain [65]. Similarly, HSV-1 glycoprotein D employs physically separable N-terminal and C-terminal regions to bind Nectin-1 and initiate membrane fusion [66]. Human Cytomegalovirus gH/gL/gO complex also presents multiple contact points across its structure to engage distinct domains of a single platelet-derived growth factor receptor *α* (PDGFR *α*) molecule [67].

The identification of two CD163-binding motifs in GP2 aligns with a broader trend emerging from PRRSV entry research: the CD163–virus interface is multivalent and structurally distributed across multiple domains rather than governed by a single dominant binding site [12]. Although SRCR5 is required for PRRSV infection, it does not fully determine susceptibility, as additional CD163 domains and structural contexts also contribute to efficient infection [28,29, 62, 68,76]. Consistent with this, expression of SRCR5 alone cannot block PRRSV infection, whereas larger CD163 fragments containing SRCR5–9 are able to confer resistance, suggesting that additional SRCR domains contribute to efficient viral recognition [77,78]. Antibody-blocking studies further support this ‘Multi-Domain’ model, as monoclonal antibodies targeting different epitopes in SRCR5 or SRCR7 inhibit infection, indicating that several CD163 regions participate in viral engagement [79]. Our recent *in vivo* findings that pigs lacking CD163 PSTII-domain-coding exon 13 are resistant to PRRSV infection provide additional support for a multi-site receptor interface [76]. In this context, the two conserved viral contact regions we defined within GP2 offer a mechanistic framework for interpreting these functional observations. Together, these data suggest that PRRSV recognizes CD163 through multiple, spatially proximal contact points – an interpretation consistent with structural predictions placing SRCR domains implicated in PRRSV susceptibility in close proximity within the 3D architecture of CD163 [12,76]. By revealing dual viral interaction motifs within GP2, our work further strengthens the Multi-Domain model and provides a foundation for designing targeted inhibitors, such as peptides or molecular mimetics, capable of disrupting GP2–CD163 engagement at multiple receptor sites.

Although our mapping identified two discrete CD163-binding regions within the ectodomain – residues 88–120 and 176–196 – our data also indicate that additional parts of GP2 may indirectly affect receptor engagement. Specifically, deletion of the TM and cytoplasmic domains severely impaired CD163 binding by the SP-129-256 construct, suggesting that these regions help maintain proper folding and presentation of the C-terminal ectodomain. This raises the possibility that residues outside the two minimal binding motifs, including residue 160 that influences PRRSV-2 tropism [34], may contribute to CD163 recognition by stabilizing the ectodomain architecture or influencing long-range interactions. Thus, while residue 160 is not part of the mapped CD163-contact motifs, its previously reported role in tropism may reflect structural or conformational effects rather than direct receptor binding.

Importantly, residue I118 in GP2a, a determinant of CD163-mediated attachment in PRRSV-2 [64], lies within the 88–120 region that we identified as essential for association with CD163. Thus, our deletion analysis captures the same functional segment implicated by fine-scale mutagenesis, reinforcing the biological relevance of this region. However, in our alignment of seven representative PRRSV-2 isolates, I118 was not conserved, suggesting that the requirement for this residue may be strain-specific rather than universal. This interpretation is consistent with a recent evolutionary analysis of PRRSV-2 GP2 sequences from China (1996–2023), which reported frequent amino-acid substitutions at site 118 among lineage 1 strains [80]. In addition, residues 91, 97 and 98 – previously shown to influence MARC-145 adaptation through amino-acid substitutions – also fall within the region we mapped [32,33]. Notably, residues 97 and 98 were fully conserved in our alignment, and residue 91 showed partial conservation (T/V), indicating selective pressure within this sub-region. Together, these findings suggest that although I118 may enhance CD163 interaction in strains that encode it, the broader 88–120 interval likely contains additional conserved determinants that support CD163 binding across PRRSV-2 lineages. This interpretation is consistent with our co-IP data, in which mutants 1–96 and 1–110 retained measurable – but reduced – interaction with CD163 compared with mutant 1–120. Consistent with this notion, removal of residue 118 reduced, but did not abolish, GP2 association with CD163 [64].

Our findings also integrate well with recent reports showing that host restriction factors specifically target GP2 as a vulnerability in the PRRSV life cycle [81]. Both DDX27 and NLRP12 negatively regulate PRRSV by promoting GP2 degradation through autophagy- and MARCH8-mediated ubiquitination pathways, respectively [35,36]. Together with our demonstration that the GP2 SP and the 88–120 and 176–196 ectodomain regions are essential for CD163 engagement and infection, these observations support a model in which GP2 occupies a strategic position at the intersection of viral entry and host immune control. Thus, targeting GP2 function – whether through host factors, neutralizing antibodies or small molecules – may represent an especially effective strategy for antiviral intervention.

Finally, the strong functional constraints implied by the conservation of key residues within the CD163-binding regions across the PRRSV-2 isolates examined suggest that these motifs may not readily tolerate variation without incurring a fitness cost. This possibility is particularly appealing from a vaccine and therapeutic perspective, as conserved GP2 surfaces required for CD163 engagement could represent broadly relevant targets. Although our sequence analysis was limited to a subset of PRRSV-2 strains, future work should assess the evolutionary conservation of these GP2 determinants across the full breadth of PRRSV-2 lineages, as well as PRRSV-1 and other arteriviruses, and determine whether antibodies or small molecules directed against these regions can block infection in primary macrophages or *in vivo*.

In summary, we show that the GP2 SP is cleaved and is both necessary and sufficient for ER localization, that SP integrity and cleavage are required for GP2 maturation, glycoprotein complex assembly and viral replication, and that two conserved GP2 ectodomain regions (residues 88–120 and 176–196) are essential for CD163 interaction and PRRSV-2 infection. These findings refine our understanding of the molecular interface between PRRSV and CD163, and position GP2 as an attractive target for next-generation antiviral strategies and rational vaccine design.

## Supplementary material

10.1099/jgv.0.002287 Supplementary Material 1.

## Abbreviations

co-IP: co-immunoprecipitation
DMEM: Dulbecco’s modified Eagle’s medium
Endo H: endoglycosidase H
ER: endoplasmic reticulum
ERGIC: ER/ER–Golgi intermediate compartment
gK: glycoprotein K
G38V: glycine 38 with valine
hpi: hours post-infection
HSV-1: herpes simplex virus 1
IP: immunoprecipitation
PAM: porcine alveolar macrophage
PNGase F: Peptide-N-glycosidase F
PRRSV: porcine reproductive and respiratory syndrome virus
SP: signal peptide
SPP: signal peptide peptidase
SRCR: scavenger receptor cysteine-rich
TM: transmembrane
WB: western blotting
WCE: whole-cell extract

## References

[1] Holtkamp D, Kliebenstein J, Neumann E, Zimmerman J, Rotto H (2013). Assessment of the economic impact of porcine reproductive and respiratory syndrome virus on United States pork producers. J Swine Health Prod.

[2] Cavanagh D (1997). Nidovirales: a new order comprising coronaviridae and arteriviridae. *Arch Virol*.

[3] Snijder EJ, Meulenberg JJ (1998). The molecular biology of arteriviruses. J Gen Virol.

[4] King AMQ, Lefkowitz EJ, Mushegian AR, Adams MJ, Dutilh BE (2018). Changes to taxonomy and the International Code of Virus Classification and Nomenclature ratified by the International Committee on Taxonomy of Viruses (2018). Arch Virol.

[5] Brinton MA, Gulyaeva AA, Balasuriya UBR, Dunowska M, Faaberg KS (2021). ICTV virus taxonomy profile: arteriviridae 2021. J Gen Virol.

[6] Kuhn JH, Lauck M, Bailey AL, Shchetinin AM, Vishnevskaya TV (2016). Reorganization and expansion of the nidoviral family arteriviridae. Arch Virol.

[7] Neumann EJ, Kliebenstein JB, Johnson CD, Mabry JW, Bush EJ (2005). Assessment of the economic impact of porcine reproductive and respiratory syndrome on swine production in the United States. J Am Vet Med Assoc.

[8] Duan X, Nauwynck HJ, Pensaert MB (1997). Virus quantification and identification of cellular targets in the lungs and lymphoid tissues of pigs at different time intervals after inoculation with porcine reproductive and respiratory syndrome virus (PRRSV). Vet Microbiol.

[9] Law SKA, Micklem KJ, Shaw JM, Zhang X, Dong Y (1993). A new macrophage differentiation antigen which is a member of the scavenger receptor superfamily. Eur J Immunol.

[10] Ritter M, Buechler C, Langmann T, Schmitz G (1999). Genomic organization and chromosomal localization of the human CD163 (M130) gene: a member of the scavenger receptor cysteine-rich superfamily. Biochem Biophys Res Commun.

[11] Su C-M, Rowland RRR, Yoo D (2021). Recent advances in PRRS virus receptors and the targeting of receptor-ligand for control. Vaccines.

[12] Rowland RRR, Brandariz-Nuñez A (2024). Role of CD163 in PRRSV infection. Virology.

[13] Ye N, Wang B, Feng W, Tang D, Zeng Z (2022). PRRS virus receptors and an alternative pathway for viral invasion. Virus Res.

[14] Li R, Qiao S, Zhang G (2022). Reappraising host cellular factors involved in attachment and entry to develop antiviral strategies against porcine reproductive and respiratory syndrome virus. Front Microbiol.

[15] Whitworth KM, Rowland RRR, Ewen CL, Trible BR, Kerrigan MA (2016). Gene-edited pigs are protected from porcine reproductive and respiratory syndrome virus. Nat Biotechnol.

[16] Yu P, Wei R, Dong W, Zhu Z, Zhang X (2019). CD163ΔSRCR5 MARC-145 cells resist PRRSV-2 infection via inhibiting virus uncoating, which requires the interaction of CD163 with calpain 1. Front Microbiol.

[17] Kappes MA, Faaberg KS (2015). PRRSV structure, replication and recombination: Origin of phenotype and genotype diversity. Virology.

[18] Lunney JK, Fang Y, Ladinig A, Chen N, Li Y (2016). Porcine Reproductive and Respiratory Syndrome Virus (PRRSV): pathogenesis and interaction with the immune system. Annu Rev Anim Biosci.

[19] Han M, Yoo D (2014). Engineering the PRRS virus genome: updates and perspectives. Vet Microbiol.

[20] Stoian AMM, Rowland RRR (2019). Challenges for Porcine Reproductive and Respiratory Syndrome (PRRS) vaccine design: reviewing virus glycoprotein interactions with CD163 and targets of virus neutralization. Vet Sci.

[21] Veit M, Matczuk AK, Sinhadri BC, Krause E, Thaa B (2014). Membrane proteins of arterivirus particles: structure, topology, processing and function. Virus Res.

[22] Wissink EHJ, Kroese MV, van Wijk HAR, Rijsewijk FAM, Meulenberg JJM (2005). Envelope protein requirements for the assembly of infectious virions of porcine reproductive and respiratory syndrome virus. J Virol.

[23] Das PB, Vu HLX, Dinh PX, Cooney JL, Kwon B (2011). Glycosylation of minor envelope glycoproteins of porcine reproductive and respiratory syndrome virus in infectious virus recovery, receptor interaction, and immune response. Virology.

[24] Meulenberg JJ, Petersen-den Besten A, De Kluyver EP, Moormann RJ, Schaaper WM (1995). Characterization of proteins encoded by ORFs 2 to 7 of lelystad virus. Virology.

[25] Wei Z, Tian D, Sun L, Lin T, Gao F (2012). Influence of N-linked glycosylation of minor proteins of porcine reproductive and respiratory syndrome virus on infectious virus recovery and receptor interaction. Virology.

[26] Rowland RRR, Brandariz-Nuñez A (2024). Role of N-linked glycosylation in porcine reproductive and respiratory syndrome virus (PRRSV) infection. J Gen Virol.

[27] Das PB, Dinh PX, Ansari IH, de Lima M, Osorio FA (2010). The minor envelope glycoproteins GP2a and GP4 of porcine reproductive and respiratory syndrome virus interact with the receptor CD163. J Virol.

[28] Stoian AMM, Rowland RRR, Brandariz-Nuñez A (2022). Mutations within scavenger receptor cysteine-rich (SRCR) protein domain 5 of porcine CD163 involved in infection with porcine reproductive and respiratory syndrome virus (PRRS). J Gen Virol.

[29] Stoian AMM, Rowland RRR, Brandariz-Nuñez A (2022). Identification of CD163 regions that are required for porcine reproductive and respiratory syndrome virus (PRRSV) infection but not for binding to viral envelope glycoproteins. Virology.

[30] Tian D, Wei Z, Zevenhoven-Dobbe JC, Liu R, Tong G (2012). Arterivirus minor envelope proteins are a major determinant of viral tropism in cell culture. J Virol.

[31] Zhang H-L, Tang Y-D, Liu C-X, Xiang L-R, Zhang W-L (2018). Adaptions of field PRRSVs in Marc-145 cells were determined by variations in the minor envelope proteins GP2a-GP3. Vet Microbiol.

[32] Chen Y, Huo Z, Jiang Q, Qiu Z, Shao Z (2024). The significance of the 98th amino acid in GP2a for porcine reproductive and respiratory syndrome virus adaptation in Marc-145 cells. Viruses.

[33] Qiu M, Li S, Li S, Sun Z, Lin H (2025). The GP2a 91/97/98 amino acid substitutions play critical roles in determining PRRSV tropism and infectivity but do not affect immune responses. J Virol.

[34] Chaudhari J, Leme RA, Durazo-Martinez K, Sillman S, Workman AM (2022). A single amino acid substitution in porcine reproductive and respiratory syndrome virus glycoprotein 2 significantly impairs its infectivity in macrophages. Viruses.

[35] Chen B, Luo Y, Chen Y, Wang J, Yan J (2025). The DEAD-box RNA helicase 27 negatively regulates the replication of porcine reproductive and respiratory syndrome virus by mediating GP2a autophagy degradation and inducing interferon-β production. Front Immunol.

[36] Jing H, Song Y, Duan E, Liu J, Ke W (2024). NLRP12 inhibits PRRSV-2 replication by promoting GP2a degradation via MARCH8. Vet Microbiol.

[37] Bai Y-Z, Sun Y, Liu Y-G, Zhang H-L, An T-Q (2024). Minor envelope proteins from GP2a to GP4 contribute to the spread pattern and yield of type 2 PRRSV in MARC-145 cells. Front Cell Infect Microbiol.

[38] Lee C, Calvert JG, Welch S-KW, Yoo D (2005). A DNA-launched reverse genetics system for porcine reproductive and respiratory syndrome virus reveals that homodimerization of the nucleocapsid protein is essential for virus infectivity. Virology.

[39] Chand RJ (2013). Study of Recombination in Porcine Reproductive and Respiratory Syndrome Virus (PRRSV) Using a Novel in-Vitro System: Kansas State University.

[40] Rowland R, Brandariz-Nuñez A (2021). Analysis of the role of n-linked glycosylation in cell surface expression, function, and binding properties of SARS-CoV-2 receptor ACE2. Microbiol Spectr.

[41] Li X, Yeung DF, Fiegen AM, Sodroski J (2011). Determinants of the higher order association of the restriction factor TRIM5α and other tripartite motif (TRIM) proteins. J Biol Chem.

[42] Guo J, Liu Z, Tong X, Wang Z, Xu S (2021). Evolutionary dynamics of type 2 porcine reproductive and respiratory syndrome virus by whole-genome analysis. Viruses.

[43] Emanuelsson O, Brunak S, von Heijne G, Nielsen H (2007). Locating proteins in the cell using targetp, signalp and related tools. Nat Protoc.

[44] Käll L, Krogh A, Sonnhammer ELL (2004). A combined transmembrane topology and signal peptide prediction method. J Mol Biol.

[45] Kall L, Krogh A, Sonnhammer ELL (2007). Advantages of combined transmembrane topology and signal peptide prediction--the phobius web server. Nucleic Acids Res.

[46] von Heijne G (1983). Patterns of amino acids near signal-sequence cleavage sites. Eur J Biochem.

[47] Hegde RS, Bernstein HD (2006). The surprising complexity of signal sequences. Trends Biochem Sci.

[48] Zhang M, Veit M (2018). Differences in signal peptide processing between GP3 glycoproteins of arteriviridae. Virology.

[49] Lee C, Yoo D (2006). The small envelope protein of porcine reproductive and respiratory syndrome virus possesses ion channel protein-like properties. Virology.

[50] Barrett CT, Dutch RE (2020). Viral membrane fusion and the transmembrane domain. Viruses.

[51] Kapp K, Schrempf S, Lemberg MK, Dobberstein B (2009). Post-Targeting Functions of Signal Peptides. Protein Transport into the Endoplasmic Reticulum.

[52] Bai Y-Z, Wang S, Sun Y, Liu Y-G, Zhang H-L (2025). The full-length nsp2 replicase contributes to viral assembly in highly pathogenic PRRSV-2. J Virol.

[53] Allen SJ, Mott KR, Matsuura Y, Moriishi K, Kousoulas KG (2014). Binding of HSV-1 glycoprotein K (gK) to signal peptide peptidase (SPP) is required for virus infectivity. PLoS One.

[54] Allen SJ, Mott KR, Ghiasi H (2014). Inhibitors of signal peptide peptidase (SPP) affect HSV-1 infectivity in vitro and in vivo. Exp Eye Res.

[55] Wang S, Ghiasi H (2019). Absence of signal peptide peptidase, an essential herpes simplex virus 1 glycoprotein K Binding Partner, Reduces Virus Infectivity *In Vivo*. J Virol.

[56] Wang S, Jaggi U, Yu J, Ghiasi H (2021). Blocking HSV-1 glycoprotein K binding to signal peptide peptidase reduces virus infectivity in vitro and in vivo. PLoS Pathog.

[57] Dobrica M-O, Lazar C, Branza-Nichita N (2020). N-Glycosylation and N-Glycan processing in HBV biology and pathogenesis. Cells.

[58] Werr M, Prange R (1998). Role for calnexin and N-linked glycosylation in the assembly and secretion of hepatitis B virus middle envelope protein particles. J Virol.

[59] Shi X, Elliott RM (2004). Analysis of N-linked glycosylation of hantaan virus glycoproteins and the role of oligosaccharide side chains in protein folding and intracellular trafficking. J Virol.

[60] Wang B, Wang Y, Frabutt DA, Zhang X, Yao X (2017). Mechanistic understanding of *N*-glycosylation in Ebola virus glycoprotein maturation and function. J Biol Chem.

[61] Welch S-KW, Jolie R, Pearce DS, Koertje WD, Fuog E (2004). Construction and evaluation of genetically engineered replication-defective porcine reproductive and respiratory syndrome virus vaccine candidates. Vet Immunol Immunopathol.

[62] Van Gorp H, Van Breedam W, Van Doorsselaere J, Delputte PL, Nauwynck HJ (2010). Identification of the CD163 protein domains involved in infection of the porcine reproductive and respiratory syndrome virus. J Virol.

[63] Van Gorp H, Van Breedam W, Delputte PL, Nauwynck HJ (2009). The porcine reproductive and respiratory syndrome virus requires trafficking through CD163-positive early endosomes, but not late endosomes, for productive infection. Arch Virol.

[64] Liu G, Huang X, Yang Y, Chen M, Tian X (2025). GP2a I118 and GP4 D43 play critical roles in the attachment of PRRSV to the CD163 receptor: implications for anti-PRRSV infection targets. J Virol.

[65] Rey MA, Prasad R, Tailor CS (2008). The C domain in the surface envelope glycoprotein of subgroup C feline leukemia virus is a second receptor-binding domain. Virology.

[66] Zhou G, Roizman B (2007). Separation of receptor-binding and profusogenic domains of glycoprotein D of herpes simplex virus 1 into distinct interacting proteins. Proc Natl Acad Sci USA.

[67] Liu J, Vanarsdall A, Chen D-H, Chin A, Johnson D (2021). Cryo-electron microscopy structure and interactions of the human cytomegalovirus gHgLgO trimer with platelet-derived growth factor receptor alpha. mBio.

[68] Guo C, Wang M, Zhu Z, He S, Liu H (2019). Highly efficient generation of pigs harboring a partial deletion of the CD163 SRCR5 domain, which are fully resistant to porcine reproductive and respiratory syndrome virus 2 infection. Front Immunol.

[69] Burkard C, Lillico SG, Reid E, Jackson B, Mileham AJ (2017). Precision engineering for PRRSV resistance in pigs: macrophages from genome edited pigs lacking CD163 SRCR5 domain are fully resistant to both PRRSV genotypes while maintaining biological function. PLoS Pathog.

[70] Wang H, Shen L, Chen J, Liu X, Tan T (2019). Deletion of *CD163* Exon 7 confers resistance to highly pathogenic porcine reproductive and respiratory viruses on pigs. Int J Biol Sci.

[71] Burger BT, Beaton BP, Campbell MA, Brett BT, Rohrer MS (2024). Generation of a commercial-scale founder population of porcine reproductive and respiratory syndrome virus resistant pigs using CRISPR-cas. CRISPR J.

[72] Nesbitt C, Galina Pantoja L, Beaton B, Chen C-Y, Culbertson M (2024). Pigs lacking the SRCR5 domain of CD163 protein demonstrate heritable resistance to the PRRS virus and no changes in animal performance from birth to maturity. Front Genome Ed.

[73] Burkard C, Opriessnig T, Mileham AJ, Stadejek T, Ait-Ali T (2018). Pigs lacking the scavenger receptor cysteine-rich domain 5 of CD163 are resistant to porcine reproductive and respiratory syndrome virus 1 infection. J Virol.

[74] Wells KD, Bardot R, Whitworth KM, Trible BR, Fang Y (2017). Replacement of porcine CD163 scavenger receptor cysteine-rich domain 5 with a CD163-like homolog confers resistance of pigs to genotype 1 but not genotype 2 porcine reproductive and respiratory syndrome virus. J Virol.

[75] Chen J, Wang H, Bai J, Liu W, Liu X (2019). Generation of pigs resistant to highly pathogenic-porcine reproductive and respiratory syndrome virus through gene editing of *CD163*. Int J Biol Sci.

[76] Salgado B, Rivas RB, Pinto D, Sonstegard TS, Carlson DF (2024). Genetically modified pigs lacking CD163 PSTII-domain-coding exon 13 are completely resistant to PRRSV infection. Antiviral Res.

[77] Chen Y, Guo R, He S, Zhang X, Xia X (2014). Additive inhibition of porcine reproductive and respiratory syndrome virus infection with the soluble sialoadhesin and CD163 receptors. Virus Res.

[78] Han G, Yang H, Xu H, Zheng S, Li Y (2022). Broad antiviral peptides against PRRSV based on novel linear epitopes on porcine CD163. Int J Biol Macromol.

[79] Xu H, Liu Z, Zheng S, Han G, He F (2020). CD163 antibodies inhibit PRRSV infection via receptor blocking and transcription suppression. Vaccines.

[80] Liu K, Lv C, He C, Pang J, Lai C (2025). Analysis of the genetic evolution and recombination of the PRRSV-2 GP2 protein in China from 1996 to 2023. Microbiol Spectr.

[81] Rowland RRR, Brandariz-Nuñez A (2026). Role of viral glycoprotein 2a (GP2a) in PRRSV infection. Virology.

[82] Katoh K, Standley DM (2013). MAFFT multiple sequence alignment software version 7: improvements in performance and usability. Mol Biol Evol.

