# Supplementary material for: Signal peptide cleavage and ectodomain regions of GP2 are required for PRRSV infection
Source: J Gen Virol. 2026 Jun 18;107(6):002287. doi: 10.1099/jgv.0.002287 (PMC13278558; doi:10.1099/jgv.0.002287)
Supplement: Supplementary Material 1. [file jgv-107-02287-s001.pdf]

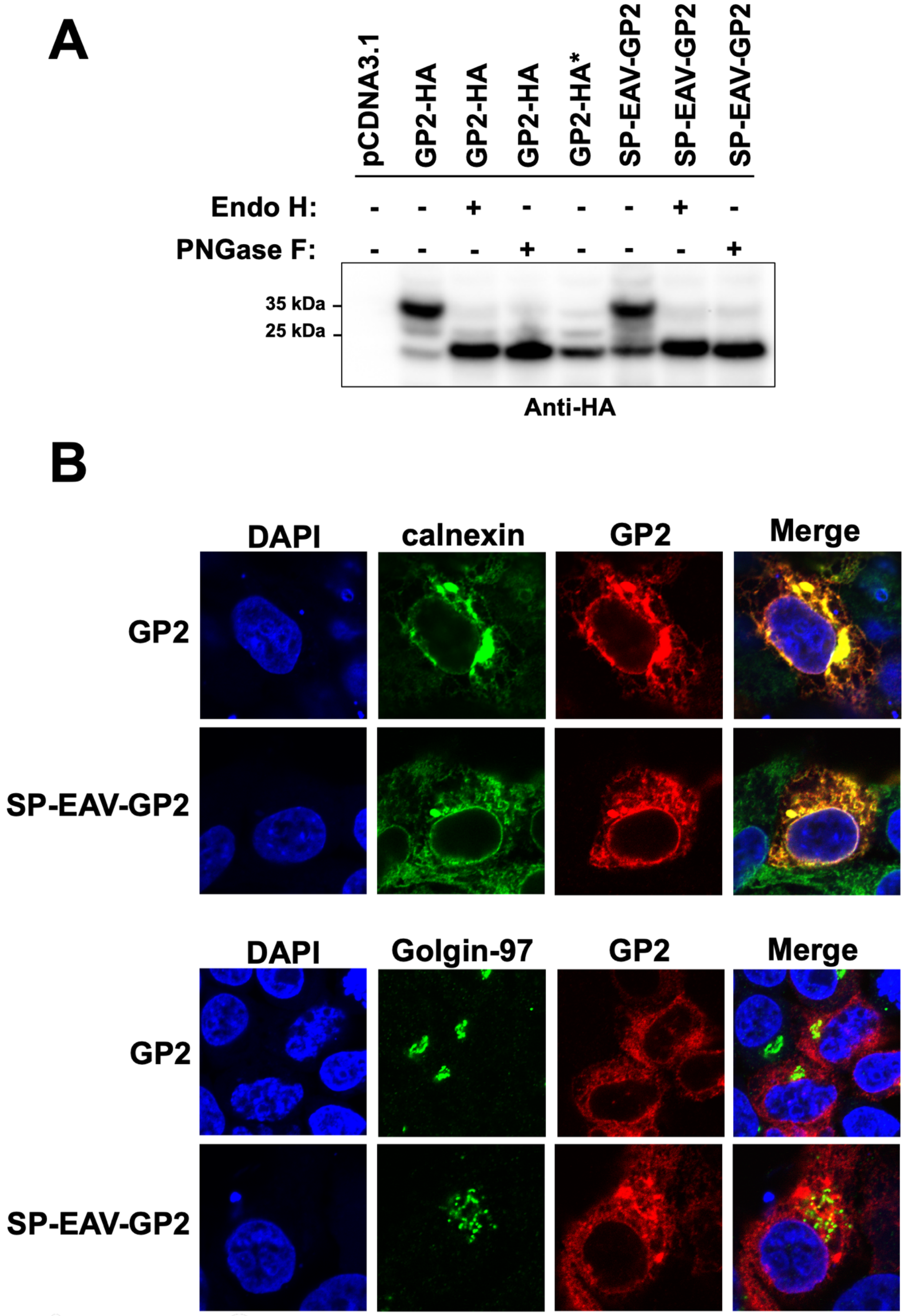
A
pCDNA3.1
GP2-HA
GP2-HA
GP2-HA
GP2-HA*
SP-EAV-GP2
SP-EAV-GP2
SP-EAV-GP2
Endo H: - - + - - - + -
PNGase F: - - - + - - - +
35 kDa
25 kDa
Anti-HA
B
DAPI
calnexin
GP2
Merge
GP2
SP-EAV-GP2
DAPI
Golgin-97
GP2
Merge
GP2
SP-EAV-GP2

Supplemental Figure 1. Replacement of the SP of PRRSV GP2 with the SP from EAV does not affect the N-glycosylation profile or intracellular localization of PRRSV GP2. (A) HEK293T cells were transfected with plasmids expressing the indicated GP2 mutants containing a C-terminal HA tag. Cell lysates were either left untreated or digested with Endo H or PNGase F, and analyzed by immunoblotting with an anti-HA antibody. (B) Colocalization of GP2 variants with the ER and Golgi. HEK293T cells were transfected with plasmids expressing the indicated GP2 mutants containing a C-terminal HA tag. After 24 h of incubation, cells were fixed and stained with an anti-HA antibody (red). The ER was visualized using an anti-calnexin antibody (green), and the Golgi apparatus was stained with an anti-Golgin-97 antibody (green). Nuclei were counterstained with DAPI (blue). Representative merged images showing colocalization (yellow) are shown.

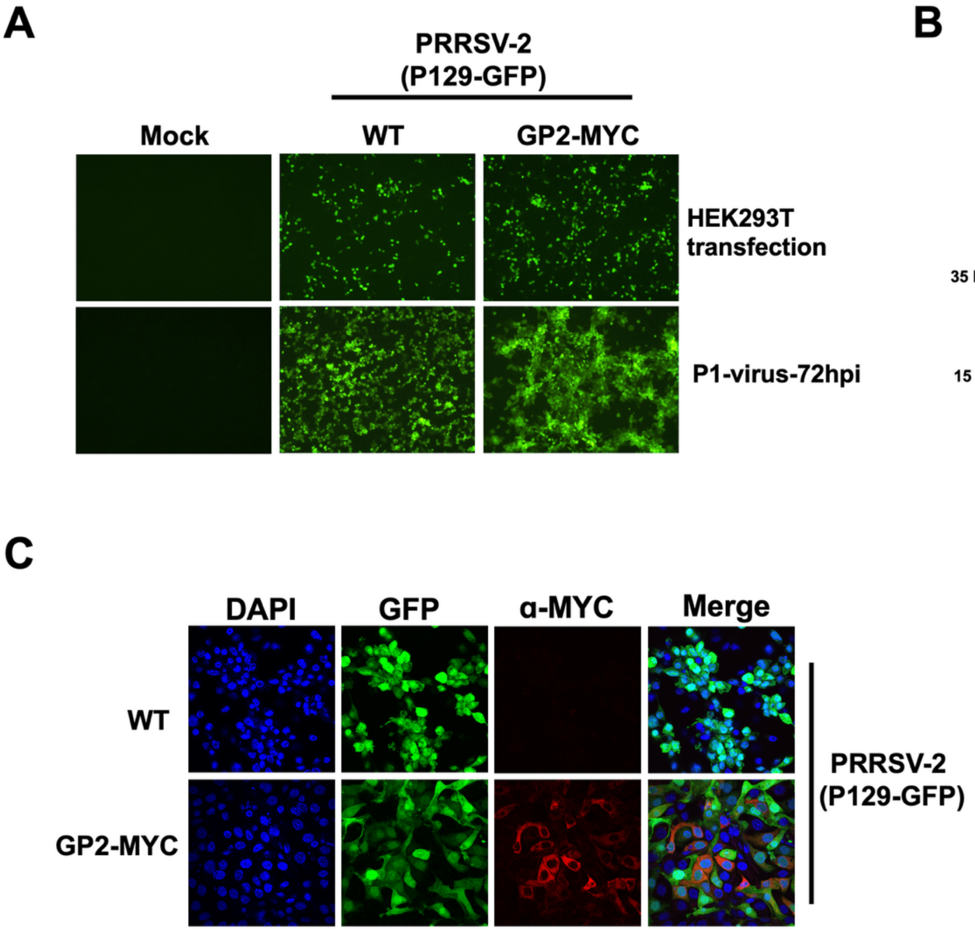

Supplemental Figure 2. Characterization of recombinant PRRSV-2 expressing GP2-MYC. (A) Supernatants from HEK293T cells transfected with PRRSV-2 (P129-GFP) cDNA clones (upper panels) were used to infect MARC-145 cells. At 72 h postinfection (hpi), infected cells were fixed and visualized under fluorescence microscopy (lower panels). (B) Infected MARC-145 cells were lysed at 72 hpi and analyzed by SDS–PAGE and Western blotting with anti-MYC antibody. Anti-N protein and anti-GAPDH antibodies served as infection and loading controls, respectively. Mock: uninfected cells. (C) MARC-145 cells infected as described above were fixed, permeabilized, and stained with anti-MYC antibody (red). Nuclei were counterstained with DAPI (blue), and GFP fluorescence was visualized directly.

Supplementary Table 1. Primers used for the construction of plasmids.

Construct	Primer Sequence
SP-GP2*	F-AATAAAATGGCCAAAAATATAATGATATCAACAATG<br>R-TGTTCTCACCATCGCCGGTTG
ΔSP-GP2	F-GCTTCACCATCGCCGGTTGGCTGGTG<br>R-CATGGTGGCGAATTCGGGCCT
GP2-MYC	F-TCGGAAGAAGACCTCTAAGCGGCCGCGGGG<br>R-TATCAATTTCTGTTCGCCGGTACCTCGAGA
SP-GP2-MYC*	F-AATAAAATGGCCAAAAATATAATGATATCAACAATG<br>R-TGTTCTCACCATCGCCGGTTG
GP2 (1-78)	F-TCTCGAGGTACCGGCTACCCA<br>R-AAAGGCCTCATAAGATCTTC
GP2 (1-81)	F-TCTCGAGGTACCGGCTACCCA<br>R-TTGGCACTGGGAAAGAAAG
GP2 (1-88)	F-TCTCGAGGTACCGGCTACCCA<br>R-TGCCAAGTGGACATTCCCACC
GP2 (1-96)	F-TCTCGAGGTACCGGCTACCCA<br>R-CCCCAAAGGATGTTTAGTTC
GP2 (1-128)	F-TAAGGCCTCTGAATTCGCCACCATGAAATGGGGTCCATGCAAAGCC<br>R-CAGAATACGTCTCGAGATTTCCAGGCAGCCTGCCCTGC
SP-129-256	F-CAGGTGGTGAGCGAGGCTACG<br>R-TGGTGAAGCCAAACAAAATGG
SP-129-196	F-TCTCGAGGTACCGGCTACCCA<br>R-GGTTGGAAAAATAGCAAACAC
SP-129-176	F-TCTCGAGGTACCGGCTACCCA<br>R-ACCTGTCATGCGCAGGTTGTGTA
GP4-V5	F-CCGAACCCGCTGCTGGGCCTGGATAGCACCTAAGCGGCCGCGGGG<br>R-AATCGGTTTGCCTGTTGCTACTGGTGGGTCGCCGGTACCTCGAGAAATTGC
GP5-FLAG	F-TCTCGAGGTACCGGCGATTACAAGGAT<br>R-ATCCTTGTAATCGCCGGTACCTCGAGAAGGG
